# Blood based hybrid nanofluid flow together with electromagnetic field and couple stresses

**DOI:** 10.1038/s41598-021-92186-z

**Published:** 2021-06-18

**Authors:** Anwar Saeed, Abdelaziz Alsubie, Poom Kumam, Saleem Nasir, Taza Gul, Wiyada Kumam

**Affiliations:** 1 Center of Excellence in Theoretical and Computational Science (TaCS-CoE), Faculty of Science, King Mongkut’s University of Technology Thonburi (KMUTT), 126 Pracha Uthit Rd., Bang Mod, Thung Khru, Bangkok, 10140 Bangkok Thailand; 2 Department of Basic Sciences, College of Science and Theoretical Studies, Saudi Electronic University, Riyadh, Saudi Arabia; 3 Center of Excellence in Theoretical and Computational Science (TaCS-CoE), Faculty of Science, King Mongkut’s University of Technology Thonburi (KMUTT), 126 Pracha Uthit Rd., Bang Mod, Thung Khru, Bangkok, 10140 Thailand; 4 Department of Medical Research, China Medical University Hospital, China Medical University, Taichung, 40402 Taiwan; 5 Department of Mathematics, City University of Science and Information Technology, Peshawar, 25000 Pakistan; 6 Applied Mathematics for Science and Engineering Research Unit (AMSERU), Program in Applied Statistics, Department of Mathematics and Computer Science, Faculty of Science and Technology, Rajamangala University of Technology Thanyaburi, Thanyaburi, Pathumthani 12110 Thailand

**Keywords:** Engineering, Mathematics and computing

## Abstract

In this investigation, heat transportation together with irreversibility analysis for the flow of couple stress hybrid nanofluid past over a stretching surface is considered. The innovative characteristics and aims of this work are to note that the transportation heat couple stress model involves EMHD, viscous dissipation, Joule heating, and heat absorption, and omission. The hybrid nanofluid is prepared due to the suspension of the solid nanoparticles of the SWCNTs and MWCNTs in pure human blood. This mathematical model is an appropriate model for biological advantages including testing of human blood for drug deliveries to various parts of the human body. Particularly, the Prandtl number used for the blood is 21 and very large as compared to the other base fluids. Necessary modifications are used to translate the defining partial differential equations and boundary conditions into a layout that can be computed. To obtain mathematical approximations for the resulting scheme of nonlinear differential equations, the innovative homotopy analysis method (HAM) is used. The explanation for velocity, energy, and entropy are exposed and the flow against various influential factors ($E,\;M,\;k,\;Q,\;S\;{\text{and}}\;Ec$) is discussed graphically. The numerical values are calculated and summarized for dimensionless $C_{{fx}} \;{\text{and}}\;Nu_{x} .$ In addition, the current study is compared for various values of $\Pr$ to that published literature and an impressive agreement in terms of finding is reported. It has also been noticed that the $M$ and $E$ factors retard the hybrid nanofluid flow, while the temperature of fluid becomes upsurges by the rise in these factors. 11.95% enhancement in the heat transfer rate has been attained using the hybrid nanofluids.

## Introduction

Nano-liquids are the colloidal collections of nanostructured materials in a base solution. Metals, oxides, carbides, and CNTs (carbon nanotubes) are widely used as nanomaterials. Water, ethylene, glycol, gasoline, and a number of other base fluids are examples. It is observed to have a variety of significant nanofluid properties, such as increased heat transfer and nanofluid stretching rate. Coolant efficacy and efficiency must be improved in a variety of fields, including communications, power generation, vehicles, engineering, and manufacturing systems, among others. Usually, nanofluids refrigerants are used to increase the performance of hydrodynamic structures. Nonliquids has recently been used in a number of applications, including aero-dynamics control production, heat exchangers, transformer freezing, chemically separated devices, water conditioning from the sun, micro-pumps, and drug recovery systems. The investigators are inspired to study the coolant, that is very efficient, because of the high specifications. As a result, the researchers want to improve the thermal capacitance of common liquids such as ethylene glycol, water, and gasoline. Regular base fluids have very poor thermal conductivity, and it is critical to increase thermal conductivity. As nanoparticles are suspended in a base fluid, their thermal performance and convective heat transport are improved. Choi^1^ initially agreed with this concept and presented a novel class of nano-liquids characterized by a relatively greater heat capacity. Eastman et al.^2^ produced a nano-liquid with Cu-nanometer-sized materials spread in base fluid (ethylene glycol) that had a good thermal stability than almost any simple base liquid like ethylene glycol. Khan and Pop^3^ proposed a new theoretical framework for the stable thermal and stream behavior of nano-liquids passing through a shrinking medium. Seth et al.^4^ investigated a convincing mathematical model that included the stagnation point MHD mixed convection flow of micro-polar nano-liquid in considerable detail. Efforts to the discussion of nanofluid flow under different conditions and various geometries are depicted in the studies like Rashidi et al.^5^, Mansoury et al.^6^, Rashid et al.^7^, Sheikholeslami^8^, Hatami et al.^9–12^.

In recent years, global ecosystem researchers and scientists have used two or potentially more nanoparticles in a typical base liquid to boost thermal conductivity and optimize heat transportation properties. Hybrid nano-liquids are a type of nanofluid that is made up of two or more distinct forms of nanoparticles fused together in a base liquid. Owing to the expense of usefulness and the potential to produce these fluids on a broad scale, researchers and scientists commonly use a two-step approach in the hybrid nanofluids setting. These nanofluids have a broad variety of applications in modern disciplines such as applied and biological sciences, agriculture production, and material sciences. Hybrid nanofluids' comparatively low cost aims to optimize heat and cooling storage thermal efficiency. As a result, the use of these fluids is much more efficient, as they are used in electric control engines, diesel engine petrol, cooling system expansion, and many other uses. Apart from that, in this study we take CNTs (carbon nanotubes) as nanoparticles. Because, SWCNTs and MWCNTs have become the most powerful components as nanomaterials due to its capability that strengthens that liquid's thermal behavior, excellent electrical conductivity, specific optical amplification, and greater tensile nature. Esfe et al.^13^ improving the dynamic characteristics of MWCNT–SiO_2_–ethylene glycol and reported that hybrid nanofluid may be more expense and efficient than nanofluid. Moghadassi et al.^14^ examine forced convection in a water base Al_2_O_3_ nanofluid and Cu + Al_2_O_3_ (hybrid nanofluid). The rate of heat transport of hybrid nano-liquid was discovered to be 4.73% better than Al_2_O_3_–water and 13.46% better than regular water. They came to the conclusion that a limited volume of Cu nanoparticles raises rate of heat transmission by 5. In the presence of MWCNT–Fe3O4 hybrid nanoparticles, Mohebbi et al.^15^ examined heat transport productivity in a rubberized stream with heating fragments. Afrand et al.^16^ investigated the rheological activity of Fe_3_O_4_–Ag HNF dependent on ethylene glycol at various temperatures. Izadi et al.^17^ executed a mathematical analysis on natural convections in Fe_3_O_4_–MWCNT dependent on water. At different temperatures, Esfe et al.^18^ explored how to increase the thermal conduction of a ethylene–glycol based DWCNT–SiO_2_ hybrid nano-liquid. For various temperatures, Asadi et al.^19^ tested the heat transport effectiveness of MWCNT + Al_2_O_3_ engine oil-based hybrid nano-liquid. In a comparative study, Iqbal et al.^20^ investigate the outline impacts of nanoparticles in SiO_2_-water based nanofluid and MoS_2_–SiO_2_ (hybrid nano-liquid) along with thermal radiation. They came to the conclusion that thermal radiations cause the heat profile to rise. Gul et al.^21^ studied the CNTs hybrid nano-liquid moving flow through a spinning disk. In the literature, a number of recent researches on the movement of hybrid nanofluids have been reported^22,23^.

Numerous manufacturing instruments, such as magneto-hydrodynamics propulsion, confinement of plasma, the liquid–metal freezing of atomic devices, electro-magnetic pumps, and magneto-hydrodynamics generators, use an electrically conducting liquids in the with magnetic field. A resistive type power called Lorentz force is created by the strong magnetic field, that regulates the flow. The rate of cooling can be regulated in heat transfer processes to produce spectacular process efficiency. The rate of liquid cooling is regulated under the effect of a superficially induced magnetic field. The analysis of MHD liquid stream is emphasized by the investigators due to its vast potential use in a variety of manufacturing and engineering problems. The researchers are moving towards studying the MHD fluid flow in order to meet the demands of the current facets of the investigation. Pavlov^24^ was the first to develop a remarkable framework for the inviscid MHD movement of a viscous liquid passing an extending membrane, and his work is remembered for its significant applications. Sheikholeslami et al.^25^ expressed a deep desire to look at mathematical simulations of magneto-hydrodynamics nano-liquid stream along with heat exchange across parallel plates in a spinning system with viscous dissipation. They addressed a series of key observations, containing the existence of the magnitude of the drag force and the rate of heat transmission in relation to a range of related parameter values. They showed that while the electromagnetic and rotating variables had good impacts on the strength of the drag force, they all had negative effects on the heat transfer rate. Khan and Makinde^26^ used MHD laminar boundary layer movement to analyze the motion of an electrically charged liquid nanofluid containing gyrotatic microorganisms over a convectively heated extending surface. The state of the convective control volume was kept in mind. With the multimedia part, Hsiao^27^ created a methodology for micropolar nanofluid flow vs a stretching/shrinking sheet of magneto-hydrodynamics and viscous dissipation effects, along with the effect of Brownian motion and the thermophoretic. The papers show helps on the subject of magneto-hydrodynamics movement of an electrical conducting liquid under various circumstances such as Krishna et al.^28^, Hayat et al.^29^ and Chaudhary and Kanika^30^.

The analysis of non-Newtonian fluids is gaining a lot of interest from scientists and researchers because of its wide variety of uses in manufacturing and engineering sectors. Casson developed a fluid flow model that included non-Newtonian liquid flow in 1995. Casson fluid is a kind of nanofluid that is essential in a variety of situations. The Casson fluid flow model has recently gained attention due to its interesting use in human life. Honey, chili sauce, jelly, and blood are also example of Casson fluid. In modern research, the Casson fluid flow model has a remarkable necessity. Casson fluid shows yield tension properties. When the yielding tension is strong sufficiently, then Casson fluid becomes a Newtonian fluid. Although strain rate is much less than shear stress, the Casson fluid starts to change. Eldabe and Salwa^31^ became the first to take note to the energy transfer of consistent MHD non-Newtonian Casson fluid flow between two co-axial tubes. It took several years for the investigation of this work to be developed. The influence of an incident particle on Casson fluid motion in two vertical dimensions flow through a porous and circular extended surface was investigated by Nadeem et al.^32^. When the Casson flow form, as well as other fluid flow parameters, were changed, they produced interesting results. Prashu and Nandkeolyar^33^ proposed a mathematical model focused on the massive parameters of Casson fluid in real life, electrically conducting magnetohydrodynamic flow of Casson fluid over the stretching layer under the combined effects of radiative heat transfer and Hall current, in order to obtain insightful results about the power of unsteady 3D incompressible fluids. Recently several scholars present a variety of related and valuable investigation such as Usman et al.^34^, Shah et al.^35^, Alkasasbeh et al.^36^ and Dero et al.^37^.

This is examined while evaluating the previously discussed publications that study on EMHD aspects of magnetized couple stress type hybrid nanofluid via entropy generation research is innovative, but also acknowledging that the couple stress model challenged bilaterally on stretching surface has not yet been studied. So, there is an ongoing attempt to bridge such a space. Overall, this research investigation is arranged according to the following manner. Initially, the model equations in the format of PDEs describing the designed momentum and heat transport and afterward transfigured these expressions by matching quantities into ODEs. Then the result of the corresponding complicated set of ODEs is computed through HAM methodology^38–41^. To identify the behavior of related factors in the flow field, graphical visualizations with physical justifications are also demonstrated in the result and discussion section. The consistency of the present study of the solution with the previous literature is also recognized for $\Pr$ and appropriate agreement is noted. Finding from the current model of flowing hybrid nanofluid are expected to be important in biomedical, technological, and different manufacturing processes.

## Mathematical modelling

**Figure 1:**
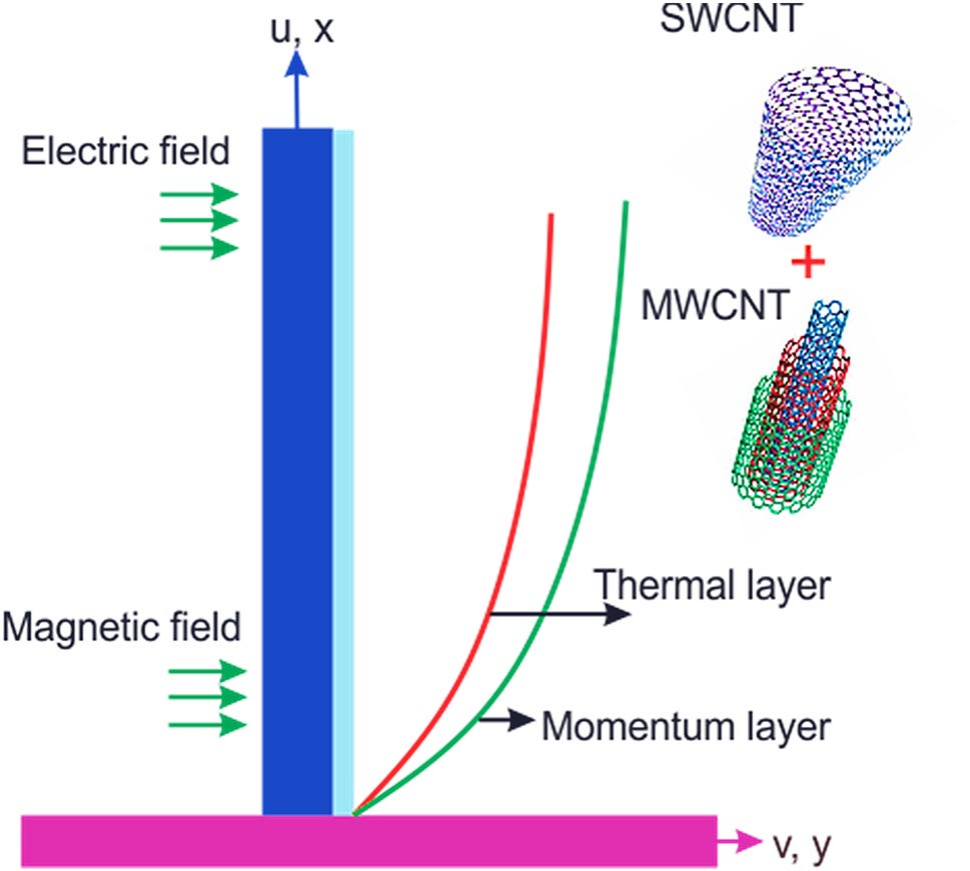
Geometry of the problem.

The flow of the electro-hydrodynamic couple stress nanofluid is considered over an extending surface. The combined efforts of the Lorentz force and electric current are counted in the flow regime. The energy expression containing the joule heating and viscous dissipation terminologies. The wall temperature denoted by $T_{w}$ for the nanofluid and the free space temperature is $T_{\infty }$. The physical structure of the fluid flow is displayed in Fig. 1.

Employing above suppositions, the modeled problem is transformed to the following form^29,36^

1 $$u_{x} + v_{y} = 0,$$

2 $$uu_{x} + vu_{y} = \upsilon _{{hnf}} u_{{yy}} + \frac{{\sigma _{hnf} }}{{\rho _{{hnf}} }}\left( {E_{0} B_{0} - B_{0}^{2} u} \right) - \frac{{\eta _{0} }}{{\rho _{{hnf}} }}u_{{yyyy}} ,$$

3 $$uT_{x} + vT_{y} = \alpha _{{hnf}} T_{{yy}} + \frac{{\mu _{{hnf}} }}{{\left( {\rho c_{p} } \right)_{{hnf}} }}u_{y}^{2} + \frac{\sigma_{{hnf}} }{{\left( {\rho c_{p} } \right)_{{hnf}} }}\left( {uB_{0} - E_{0} } \right)^{2} + \frac{{Q_{0} }}{{\left( {\rho c_{p} } \right)_{{hnf}} }}\left( {T - T_{\infty } } \right),$$

### Boundary conditions

Suitable boundary constraints are^29^

4 $$\begin{aligned} & {\text{at}}\;y = 0,\quad u = bx = u_{w} \left( x \right),\quad v = 0\quad {\text{and}}\quad T = T_{w} , \\ & {\text{at}}\;y \to \infty ,\quad u = 0 = v\quad {\text{and}}\quad T \to T_{\infty } . \\ \end{aligned}$$

where $u$ and $v$ are the velocities element acting along the x axis while $v$ acting along y-axis. $\rho _{{hnf}}$ represents hybrid nanofluid density, $E_{0}$ strength of electric field, $\nu _{{hnf}}$ (kinematic viscosity), $\alpha _{{hnf}}$ (thermal diffusivity*)*, $\left( {\rho c_{p} } \right)_{{hnf}}$ (heat capacity) and $\mu _{{hnf}}$ (dynamic viscosity) of hybrid nanofluid, $Q_{0}$ is used for the heat absorption/omission, the electric conductivity is defined by $\sigma ^{*}$.

### Similarity transformations

The similarity transformations are provided in order to simplify the current framework as

5 $$u = \left( {bx} \right)\;F^{\prime}\left( \eta \right),\quad v = \left( { - \sqrt {b\nu } } \right)F\left( \eta \right),\quad \Theta \left( \eta \right)(T_{w} - T_{\infty } ) = T - T_{\infty } ,\quad \eta = y\sqrt {\frac{b}{\nu }} ,$$

Utilizing Eq. (5) in Eqs. (1–3) to obtain the following dimensionless form of the expression

6 $$F^{\prime\prime\prime} + \frac{{\mu _{f} }}{{\mu _{{hnf}} }}\left[ {\frac{{\rho _{{hnf}} }}{{\rho _{f} }}\left( {FF^{\prime\prime} - F^{{\prime 2}} - kF^{v} + M\left( {E - F^{\prime}} \right) } \right)} \right] = 0,$$

7 $$\frac{{k_{{hnf}} }}{{k_{f} }}\Theta ^{\prime\prime } + \Pr \frac{{\left( {\rho Cp} \right)_{{hnf}} }}{{\left( {\rho Cp} \right)_{f} }}F\Theta ^{\prime } + Ec\Pr \left[ {M\left( {E - F^{\prime}} \right)^{2} + \frac{{\mu _{{hnf}} }}{{\mu _{f} }}\left( {F^{\prime\prime}} \right)^{2} } \right] + Q\Pr \Theta = 0,$$

The dimensionless boundary constraints are:

8 $$\begin{array}{*{20}l} {F\left( 0 \right) = 0,} \hfill & {F^{\prime}\left( 0 \right) = 1 = \Theta \left( 0 \right),} \hfill \\ {F\left( \infty \right) = 0,} \hfill & {\Theta \left( \infty \right) = 0.} \hfill \\ \end{array}$$

In Eqs. (6) and (7), $k$ is the couple stress constraint, Prandtl number $Pr$, $M$ is the magnetic parameter, $Q$ is the heat source/sink factor, $E$ is electric field factor and $Ec$ is the Eckert number are defined below

9 $$E = \frac{{E_{0} }}{{B_{0} u_{w} }},\;\;M = \frac{{\sigma _{f} B_{0}^{2} }}{{b\rho _{f} }},\;\;Ec = \frac{{u_{w} ^{2} }}{{c_{p} \left( {T_{w} - T_{\infty } } \right)}},\;\;\Pr = \frac{{\nu _{f} }}{{\alpha _{f} }},\;\;k = \frac{{\eta _{0} b}}{{\upsilon _{f}^{2} \rho _{f} }},\;\; Q = \frac{{Q_{0} }}{{b\left( {\rho c_{p} } \right)_{f} }}.$$

### Thermo-physical properties of HNF

The thermo-physical properties for the hybrid nano-liquids are fallows as^21^

10 $$\begin{aligned} \mu _{{hnf}} & = \frac{{\mu _{f} }}{{\left( {1 - \phi _{1} } \right)^{{2.5}} \left( {1 - \phi _{2} } \right)^{{2.5}} }} \\ \frac{{\rho _{{hnf}} }}{{\rho _{f} }} & = \left[ {\left( {1 - \phi _{2} } \right)\left\{ {1 - \left( {1 - \frac{{\rho _{{SW{\text{C}}NT}} }}{{\rho _{f} }}} \right)\phi _{1} + \phi _{2} \frac{{\rho _{{MW{\text{C}}NT}} }}{{\rho _{f} }}} \right\}} \right], \\ \frac{{\left( {\rho Cp} \right)_{{hnf}} }}{{\left( {\rho Cp} \right)_{f} }} & = \left[ {\left( {1 - \phi _{2} } \right)\left\{ {1 - \left( {1 - \frac{{\left( {\rho Cp} \right)_{{SW{\text{C}}NT}} }}{{\left( {\rho Cp} \right)_{f} }}} \right)\phi _{1} + \phi _{2} \frac{{\left( {\rho Cp} \right)_{{MW{\text{C}}NT}} }}{{\left( {\rho Cp} \right)_{f} }}} \right\}} \right], \\ \frac{{k_{{hnf}} }}{{k_{{bf}} }} & = \frac{{1 - \phi _{2} + 2\phi _{2} \frac{{k_{{SWCNT}} }}{{(k_{{MCNT}} - k_{{bf}} )}} - \ln \frac{{k_{{SWCNT}} + k_{{bf}} }}{{2k_{{_{{bf}} }} }}}}{{1 - \phi _{2} + 2\phi _{2} \frac{{k_{{bf}} }}{{(k_{{SWCNT}} - k_{{bf}} )}} - \ln \frac{{k_{{SWCNT}} + k_{{bf}} }}{{2k_{{_{{bf}} }} }}}}, \\ \frac{{k_{{bf}} }}{{k_{f} }} & = \frac{{1 - \phi _{1} + 2\phi _{1} \frac{{k_{{MWCNT}} }}{{(k_{{MWCNT}} - k_{{bf}} )}} - \ln \frac{{k_{{MWCNT}} + k_{{bf}} }}{{2k_{{_{{bf}} }} }}}}{{1 - \phi _{1} + 2\phi _{1} \frac{{k_{{bf}} }}{{(k_{{MWCNT}} - k_{{bf}} )}} - \ln \frac{{k_{{MWCNT}} + k_{{bf}} }}{{2k_{{_{{bf}} }} }}}}. \\ \end{aligned}$$

### Quantities of interest

The physical quantities of curiosity like, drag force and heat flux have plentiful applications in the field of science and engineering. In the recent study blood is used as the base fluid. Therefore, the applications of these physical constraints are also very meaningful for the bio-engineering. The mathematical expressions for the drag force and heat transfer analysis are defined as:

11 $$C_{{fx}} = \frac{{\tau _{w} }}{{\frac{1}{2}\rho _{{hnf}} \left( {u_{w} } \right)^{2} }},\quad Nu_{x} = \frac{{xq_{w} }}{{k_{{hnf}} \left( {T_{w} - T_{\infty } } \right)}},$$

where $\tau _{w}$ is the shear stress and $q_{w}$ denotes heat flux nears the surface. Utilizing Eqs. (5), (10) yields

12 $$C_{{fx}} Re_{x} ^{{0.5}} = 2\left( {\frac{{\mu _{{hnf}} }}{{\mu _{f} }}} \right)F^{\prime\prime}\left( 0 \right),\quad Nu_{x} Re_{x} ^{{ - 0.5}} = - \left( {\frac{{k_{{hnf}} }}{{k_{f} }}} \right)\Theta ^{\prime } \left( 0 \right).$$

### Entropy generation rate

13 $$\begin{aligned} S_{g} & = \frac{{k_{{hnf}} }}{{T_{\infty }^{2} }}\left( {\frac{{\partial T}}{{\partial y}}} \right)^{2} + \frac{{\mu _{{hnf}} }}{{T_{\infty } }}\left( {\frac{{\partial u}}{{\partial y}}} \right)^{2} + \frac{{\sigma_{hnf} }}{{T_{\infty } }}\left( {uB_{0} - E_{0} } \right)^{2} - \eta ^{*} \left( {\frac{{\partial u}}{{\partial y}}} \right)^{4} , \\ S_{G} & = \frac{{k_{{hnf}} }}{{k_{f} }}\lambda \left( {\Theta ^{\prime } } \right)^{2} + Br\left[ {\frac{{\mu _{{hnf}} }}{{\mu _{f} }}\left( {f^{\prime\prime}} \right)^{2} + M\left( {E - F^{\prime}} \right)^{2} } \right] + Q\Theta . \\ \end{aligned}$$

Here $S_{G} = \frac{{S_{g} T_{\infty } }}{{\left( {T_{w} - T_{\infty } } \right)}}$ is the rate of entropy generation, $\lambda = \frac{{T_{w} - T_{\infty } }}{{T_{\infty } }},$ is the thermal difference parameter, and $Br\left( { = \Pr Ec} \right)$ is the Brinkman number.

### Bejan number

The analysis in the ratio of the irreversibility analysis happened because of heat transport and total irreversibility called Bejan number.

14 $$Be = \frac{{\frac{{k_{{hnf}} }}{{k_{f} }}\lambda \left( {\Theta ^{\prime } } \right)^{2} }}{{\frac{{k_{{hnf}} }}{{k_{f} }}\lambda \left( {\Theta ^{\prime } } \right)^{2} + Br\left[ {\frac{{\mu _{{hnf}} }}{{\mu _{f} }}\left( {f^{\prime\prime}} \right)^{2} + M\left( {E - F^{\prime}} \right)^{2} } \right] + Q\Theta }}.$$

## Solution by HAM

The resulting differential equations are strongly nonlinear in many physical problems. The researchers face a challenge in computing numerical or analytical results to the corresponding expression. HAM is one of the most operative computational techniques for computing the series solution of non-linear partial and ordinary differential equations. This approach can be used to solve strongly nonlinear problems without the need for a large or small variable. This approach gives us a lot of versatility in terms of selecting and changing the convergence area and estimation rate. The homotopy analysis approach has an advantage over traditional computational approaches in that it avoids rounding off errors caused by the discretization process. Furthermore, it does not necessarily require a significant volume of machine memory or time^38^. This approach has been successfully used in a variety of nonlinear scientific and technology challenges^39–41^. The transformed Eqs. (6) and (7) are solved by HAM. The trail solution is required for the HAM solution. Therefore, linear terms are selected as:

15 $$L_{{\overset{\lower0.5em\hbox{$\smash{\scriptscriptstyle\frown}$}}{F} }} (\overset{\lower0.5em\hbox{$\smash{\scriptscriptstyle\frown}$}}{F} ) = \overset{\lower0.5em\hbox{$\smash{\scriptscriptstyle\frown}$}}{f^{\prime\prime\prime}} ,\quad {\text{L}}_{{\overset{\lower0.5em\hbox{$\smash{\scriptscriptstyle\frown}$}}{\Theta } }} (\overset{\lower0.5em\hbox{$\smash{\scriptscriptstyle\frown}$}}{\Theta } ) = \overset{\lower0.5em\hbox{$\smash{\scriptscriptstyle\frown}$}}{\Theta } ^{\prime\prime}.$$

Linear operators $$L_{{\overset{\lower0.5em\hbox{$\smash{\scriptscriptstyle\frown}$}}{F} }} ,\,\text{and}\,{\text{L}}_{{\overset{\lower0.5em\hbox{$\smash{\scriptscriptstyle\frown}$}}{\Theta } }}$$ are signified as

16 $$L_{{\overset{\lower0.5em\hbox{$\smash{\scriptscriptstyle\frown}$}}{F} }} (m_{1} + m_{2} \eta + m_{3} \eta ^{2} ) = 0,\quad {\text{L}}_{{\overset{\lower0.5em\hbox{$\smash{\scriptscriptstyle\frown}$}}{\Theta } }} (m_{4} + m_{5} \eta ) = 0,$$

The non-linear operators are defined as $${\rm N}_{{\overset{\lower0.5em\hbox{$\smash{\scriptscriptstyle\frown}$}}{F} }} \,\text{and}\,\,{\rm N}_{{\overset{\lower0.5em\hbox{$\smash{\scriptscriptstyle\frown}$}}{\Theta } }} {\text{ }}$$

17 $${\text{ }}{\rm N}_{{\overset{\lower0.5em\hbox{$\smash{\scriptscriptstyle\frown}$}}{F} }} {\text{ }}\left[ {\overset{\lower0.5em\hbox{$\smash{\scriptscriptstyle\frown}$}}{F} (\eta ;\zeta )} \right] = \overset{\lower0.5em\hbox{$\smash{\scriptscriptstyle\frown}$}}{F} _{{\eta \eta \eta }} + \frac{{\mu _{f} }}{{\mu _{{hnf}} }}\frac{{\left( \rho \right)_{{hnf}} }}{{\left( \rho \right)_{f} }}\left[ {\overset{\lower0.5em\hbox{$\smash{\scriptscriptstyle\frown}$}}{F} \overset{\lower0.5em\hbox{$\smash{\scriptscriptstyle\frown}$}}{F} _{{\eta \eta }} - \overset{\lower0.5em\hbox{$\smash{\scriptscriptstyle\frown}$}}{F} _{{\eta \eta }}^{2} } \right] + \frac{{\mu _{f} }}{{\mu _{{hnf}} }}\left[ {M\left( {E - \overset{\lower0.5em\hbox{$\smash{\scriptscriptstyle\frown}$}}{F} _{\eta } } \right)^{2} - k\overset{\lower0.5em\hbox{$\smash{\scriptscriptstyle\frown}$}}{F} _{{\eta \eta \eta \eta \eta }} } \right],$$

18 $${\rm N}_{{\overset{\lower0.5em\hbox{$\smash{\scriptscriptstyle\frown}$}}{\Theta } }} \left[ {\overset{\lower0.5em\hbox{$\smash{\scriptscriptstyle\frown}$}}{F} (\eta ;\zeta ),\overset{\lower0.5em\hbox{$\smash{\scriptscriptstyle\frown}$}}{\Theta } (\eta ;\zeta )} \right] = \frac{{k_{{hnf}} }}{{k_{f} }}\overset{\lower0.5em\hbox{$\smash{\scriptscriptstyle\frown}$}}{\Theta } _{{\eta \eta }} + \Pr \frac{{\left( {\rho Cp} \right)_{{hnf}} }}{{\left( {\rho Cp} \right)_{f} }}\overset{\lower0.5em\hbox{$\smash{\scriptscriptstyle\frown}$}}{F} \overset{\lower0.5em\hbox{$\smash{\scriptscriptstyle\frown}$}}{\Theta } _{\eta } + \frac{{\mu _{{hnf}} }}{{\mu _{f} }}Ec\Pr \left( {\overset{\lower0.5em\hbox{$\smash{\scriptscriptstyle\frown}$}}{F} _{{^{{\eta \eta }} }}^{2} + M\left( {\overset{\lower0.5em\hbox{$\smash{\scriptscriptstyle\frown}$}}{E} - \overset{\lower0.5em\hbox{$\smash{\scriptscriptstyle\frown}$}}{F} _{\eta } } \right)^{2} } \right)Q\Pr \overset{\lower0.5em\hbox{$\smash{\scriptscriptstyle\frown}$}}{\Theta } ,$$

For Eqs. (6) and (7), the 0th-order problems is written as

19 $$(1 - \zeta )\,L_{{\overset{\lower0.5em\hbox{$\smash{\scriptscriptstyle\frown}$}}{F} }} \left[ {\overset{\lower0.5em\hbox{$\smash{\scriptscriptstyle\frown}$}}{F} (\eta ;\,\,\zeta ) - \,\,\overset{\lower0.5em\hbox{$\smash{\scriptscriptstyle\frown}$}}{F} _{0} (\,\eta )} \right] = p\hbar _{{\overset{\lower0.5em\hbox{$\smash{\scriptscriptstyle\frown}$}}{F} }} \,{\rm N}_{{\overset{\lower0.5em\hbox{$\smash{\scriptscriptstyle\frown}$}}{F} }} \left[ {\overset{\lower0.5em\hbox{$\smash{\scriptscriptstyle\frown}$}}{F} (\eta ;\zeta )} \right],$$

20 $$(1 - \zeta ){\text{ }}\,L_{{\overset{\lower0.5em\hbox{$\smash{\scriptscriptstyle\frown}$}}{\Theta } }} \left[ {\overset{\lower0.5em\hbox{$\smash{\scriptscriptstyle\frown}$}}{\Theta } (\eta ;\,\,\zeta ) - \overset{\lower0.5em\hbox{$\smash{\scriptscriptstyle\frown}$}}{\Theta } _{0} (\,\eta )} \right] = p\hbar _{{\overset{\lower0.5em\hbox{$\smash{\scriptscriptstyle\frown}$}}{\Theta } }} \,\,{\rm N}_{{\overset{\lower0.5em\hbox{$\smash{\scriptscriptstyle\frown}$}}{\Theta } }} \left[ {F(\eta ;\zeta ),\overset{\lower0.5em\hbox{$\smash{\scriptscriptstyle\frown}$}}{\Theta } (\eta ;\zeta )} \right],$$

While BCs are:

21 $$\begin{aligned} & \left. {\overset{\lower0.5em\hbox{$\smash{\scriptscriptstyle\frown}$}}{F} (\eta ;\zeta )} \right|_{{\eta = 0}} = 0,{\text{ }}\left. {\frac{{\partial \overset{\lower0.5em\hbox{$\smash{\scriptscriptstyle\frown}$}}{F} (\eta ;\zeta )}}{{\partial \eta }}} \right|_{{\eta = 0}} = 1,{\text{ }} \\ & \left. {\overset{\lower0.5em\hbox{$\smash{\scriptscriptstyle\frown}$}}{\Theta } (\eta ;\zeta )} \right|_{{\eta = 0}} = 1,{\text{ }} \\ & {\text{ }}\left. {\overset{\lower0.5em\hbox{$\smash{\scriptscriptstyle\frown}$}}{F} (\eta ;\zeta )} \right|_{{\eta = \infty }} \to 0,\quad \left. {\overset{\lower0.5em\hbox{$\smash{\scriptscriptstyle\frown}$}}{\Theta } (\eta ;\zeta )} \right|_{{\eta = \infty }} \to 0, \\ \end{aligned}$$

When the embedding restriction is in place $$\zeta \in [0,1]$$, to regulate the convergence of the result $$\hbar _{{\overset{\lower0.5em\hbox{$\smash{\scriptscriptstyle\frown}$}}{F} }}$$ and $$\hbar _{{\overset{\lower0.5em\hbox{$\smash{\scriptscriptstyle\frown}$}}{\theta } }}$$ are used. We take $$\zeta = 0\;{\text{and}}\;\zeta = 1$$, so,

22 $$\overset{\lower0.5em\hbox{$\smash{\scriptscriptstyle\frown}$}}{F} (\eta ;1) = \overset{\lower0.5em\hbox{$\smash{\scriptscriptstyle\frown}$}}{F} (\eta ),\quad \overset{\lower0.5em\hbox{$\smash{\scriptscriptstyle\frown}$}}{\Theta } (\eta ;1) = \overset{\lower0.5em\hbox{$\smash{\scriptscriptstyle\frown}$}}{\Theta } (\eta ),$$

By using Taylor’s series expand the $$\overset{\lower0.5em\hbox{$\smash{\scriptscriptstyle\frown}$}}{F} (\eta ;\zeta ){\text{ }}$$ and $$\overset{\lower0.5em\hbox{$\smash{\scriptscriptstyle\frown}$}}{\Theta } (\eta ;\zeta )$$ for $$\zeta = 0$$

23 $$\begin{aligned} \overset{\lower0.5em\hbox{$\smash{\scriptscriptstyle\frown}$}}{F} (\eta ;\zeta ) & = \overset{\lower0.5em\hbox{$\smash{\scriptscriptstyle\frown}$}}{F} _{0} (\eta ) + \sum\limits_{{n = 1}}^{\infty } {\overset{\lower0.5em\hbox{$\smash{\scriptscriptstyle\frown}$}}{F} _{n} (\eta )\zeta ^{n} } , \\ \overset{\lower0.5em\hbox{$\smash{\scriptscriptstyle\frown}$}}{\Theta } (\eta ;\zeta ) & = \overset{\lower0.5em\hbox{$\smash{\scriptscriptstyle\frown}$}}{\Theta } _{0} (\eta ) + \sum\limits_{{n = 1}}^{\infty } {\overset{\lower0.5em\hbox{$\smash{\scriptscriptstyle\frown}$}}{\Theta } _{n} (\eta )\zeta ^{n} } , \\ \end{aligned}$$

24 $$\overset{\lower0.5em\hbox{$\smash{\scriptscriptstyle\frown}$}}{F} _{n} (\eta ) = \left. {\frac{1}{{n!}}\frac{{\partial (\eta ;\zeta )}}{{\partial \eta }}} \right|_{{p = 0}} ,\quad \overset{\lower0.5em\hbox{$\smash{\scriptscriptstyle\frown}$}}{\Theta } _{n} (\eta ) = \left. {\frac{1}{{n!}}\frac{{\partial \overset{\lower0.5em\hbox{$\smash{\scriptscriptstyle\frown}$}}{\theta } (\eta ;\zeta )}}{{\partial \eta }}} \right|_{{p = 0}} ,$$

Now

25 $$\Re _{n}^{{\overset{\lower0.5em\hbox{$\smash{\scriptscriptstyle\frown}$}}{F} }} \left( \eta \right) = \overset{\lower0.5em\hbox{$\smash{\scriptscriptstyle\frown}$}}{F^{\prime\prime\prime}} _{{n - 1}} + \frac{{\mu _{f} }}{{\mu _{{hnf}} }}\frac{{\left( \rho \right)_{{hnf}} }}{{\left( \rho \right)_{f} }}\left[ {\sum\limits_{{j = 0}}^{{w - 1}} {\overset{\lower0.5em\hbox{$\smash{\scriptscriptstyle\frown}$}}{F} _{{w - 1 - j}} \overset{\lower0.5em\hbox{$\smash{\scriptscriptstyle\frown}$}}{F^{\prime\prime}} _{j} } - \overset{\lower0.5em\hbox{$\smash{\scriptscriptstyle\frown}$}}{F^{\prime}} _{{^{{n - 1}} }}^{2} } \right] + \frac{{\mu _{f} }}{{\mu _{{hnf}} }}\left[ {M\left( {E - F^{\prime}_{{n - 1}} } \right)^{2} - kF_{{^{{n - 1}} }}^{v} } \right] = 0,$$

26 $$\Re _{n}^{{\overset{\lower0.5em\hbox{$\smash{\scriptscriptstyle\frown}$}}{\Theta } }} (\eta ) = \frac{{k_{{hnf}} }}{{k_{f} }}\overset{\lower0.5em\hbox{$\smash{\scriptscriptstyle\frown}$}}{\Theta ^{\prime\prime}} _{{n - 1}} + \Pr \frac{{\left( {\rho Cp} \right)_{{hnf}} }}{{\left( {\rho Cp} \right)_{f} }}\sum\limits_{{j = 0}}^{{w - 1}} {\overset{\lower0.5em\hbox{$\smash{\scriptscriptstyle\frown}$}}{F} _{{w - 1 - j}} } \overset{\lower0.5em\hbox{$\smash{\scriptscriptstyle\frown}$}}{\Theta ^{\prime}} _{j} + \frac{{\mu _{{hnf}} }}{{\mu _{f} }}Ec\Pr \left( {\overset{\lower0.5em\hbox{$\smash{\scriptscriptstyle\frown}$}}{F^{\prime\prime}} _{{^{{n - 1}} }}^{2} + M\left( {E - \overset{\lower0.5em\hbox{$\smash{\scriptscriptstyle\frown}$}}{F^{\prime}} _{{n - 1}} } \right)^{2} } \right) + Q\Pr \overset{\lower0.5em\hbox{$\smash{\scriptscriptstyle\frown}$}}{\Theta } _{{n - 1}} = 0,$$

27 $${\text{While}}\;\;\chi _{n} = \left\{ {\begin{array}{*{20}l} {0,} \hfill & {{\text{if}}\;\;\zeta \le {\text{1}}} \hfill \\ {1,} \hfill & {{\text{if}}\;\;\zeta > {\text{1}}{\text{.}}} \hfill \\ \end{array} } \right.$$

## Results and discussion

**Figure 2:**
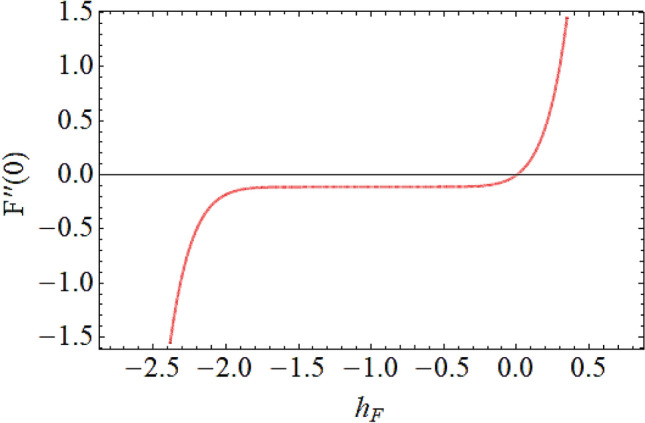
$h$ curve for velocity field.

**Figure 3:**
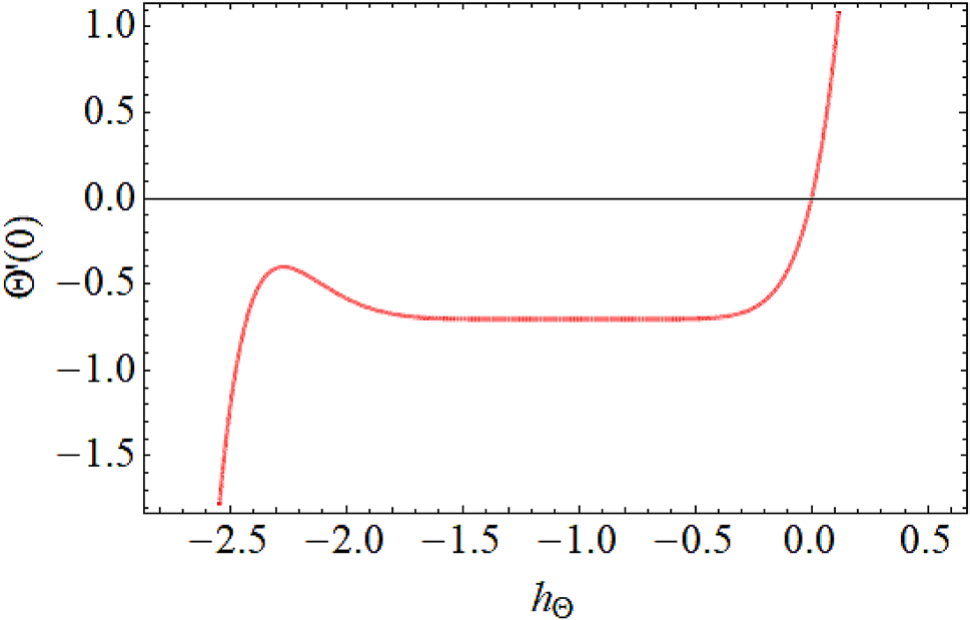
$h$ curve for temperature field.

**Figure 4:**
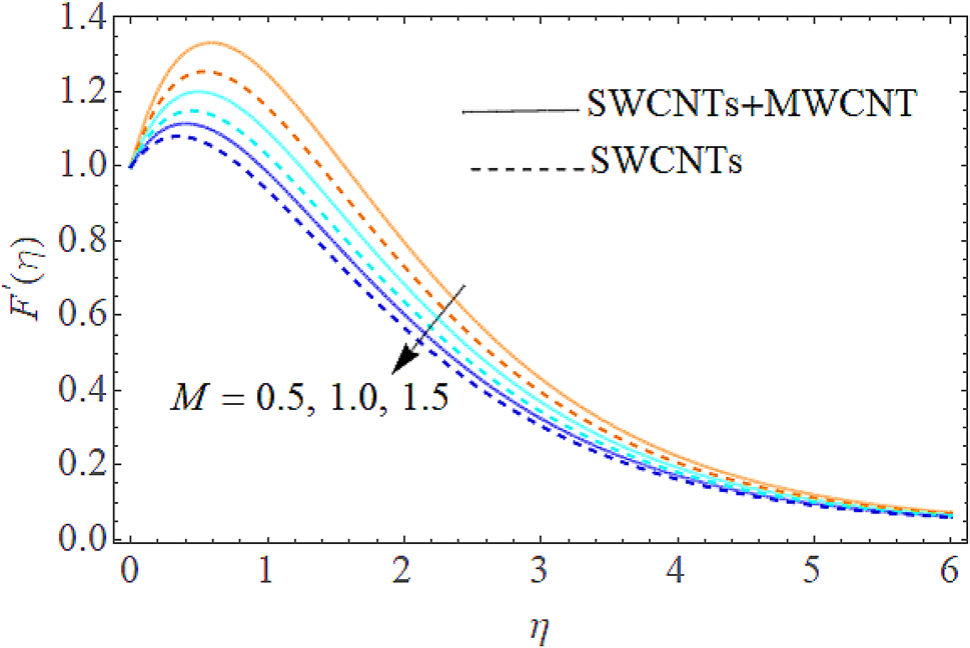
Outcome of $M$ on $F^{\prime}(\eta )$.

**Figure 5:**
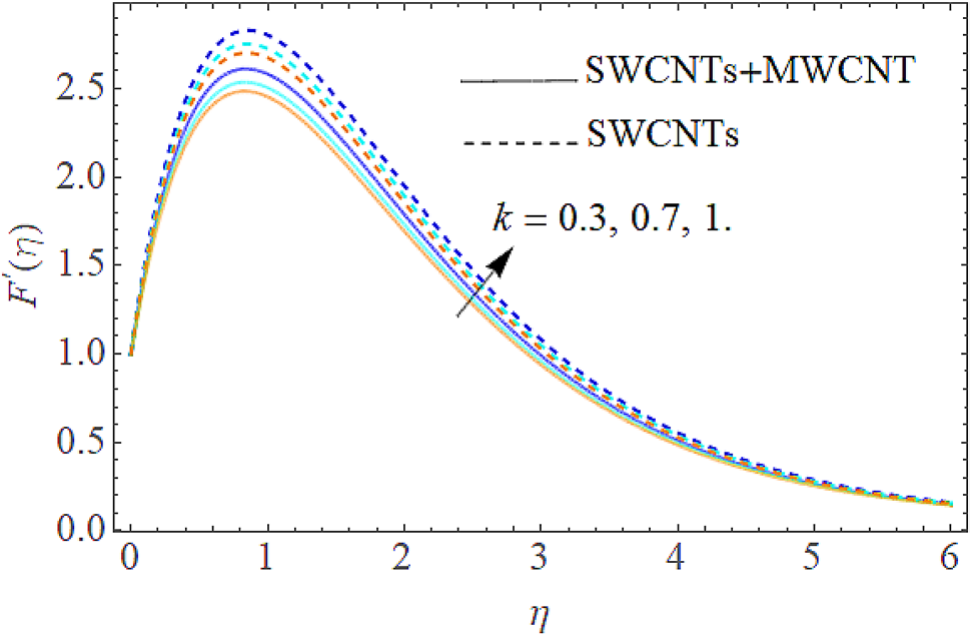
Influence of $k$ on $F^{\prime}(\eta )$.

**Figure 6:**
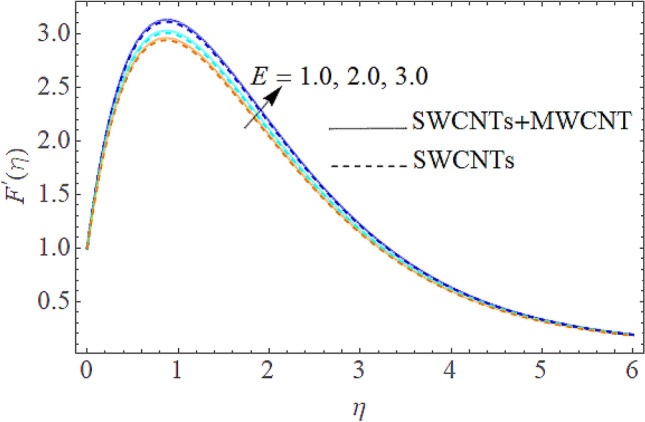
Impact of $E$ on $F^{\prime}(\eta )$.

**Figure 7:**
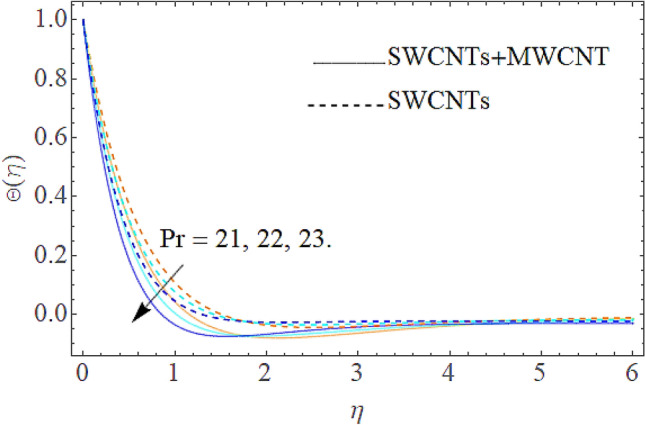
Outcome of $\Pr$ on $\Theta (\eta )$.

**Figure 8:**
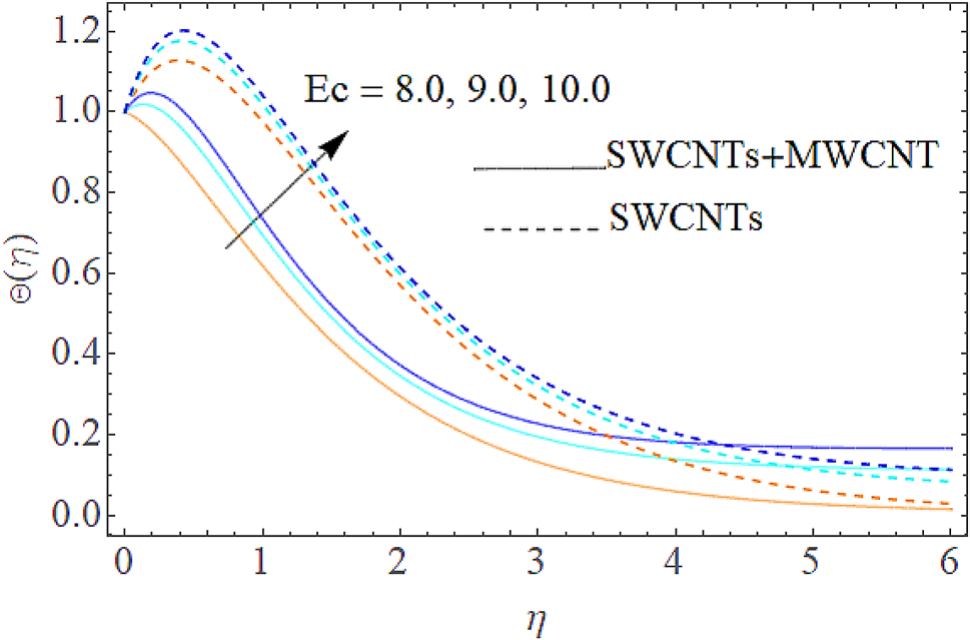
Impact of $Ec$ on $\Theta (\eta )$.

**Figure 9:**
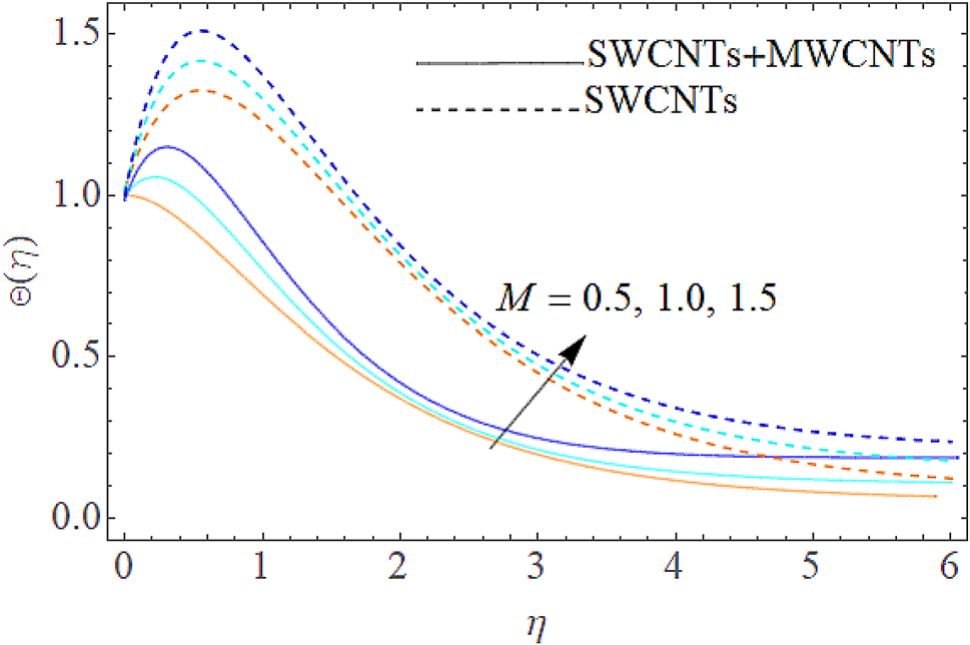
Outcome of $M$ on $\Theta (\eta )$.

**Figure 10:**
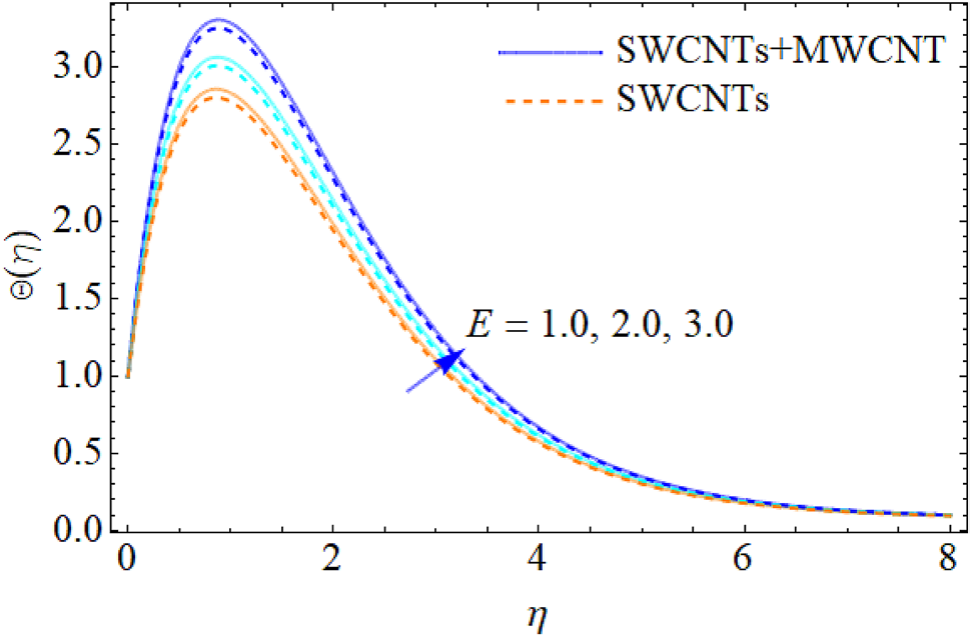
Influence of $E$ on $\Theta (\eta )$.

**Figure 11:**
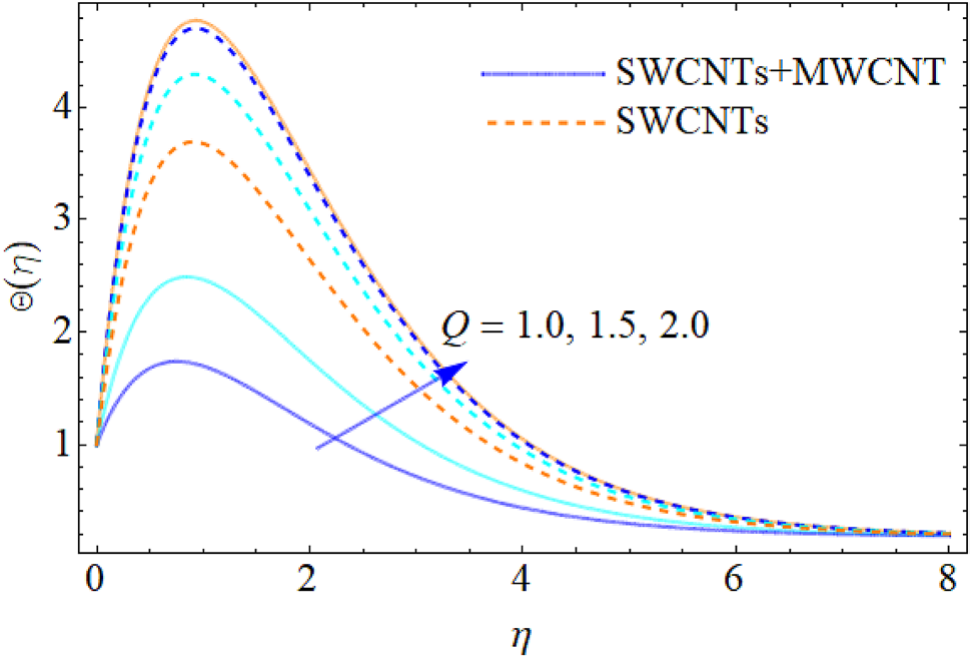
Impact of $Q$ on $\Theta (\eta )$.

**Figure 12:**
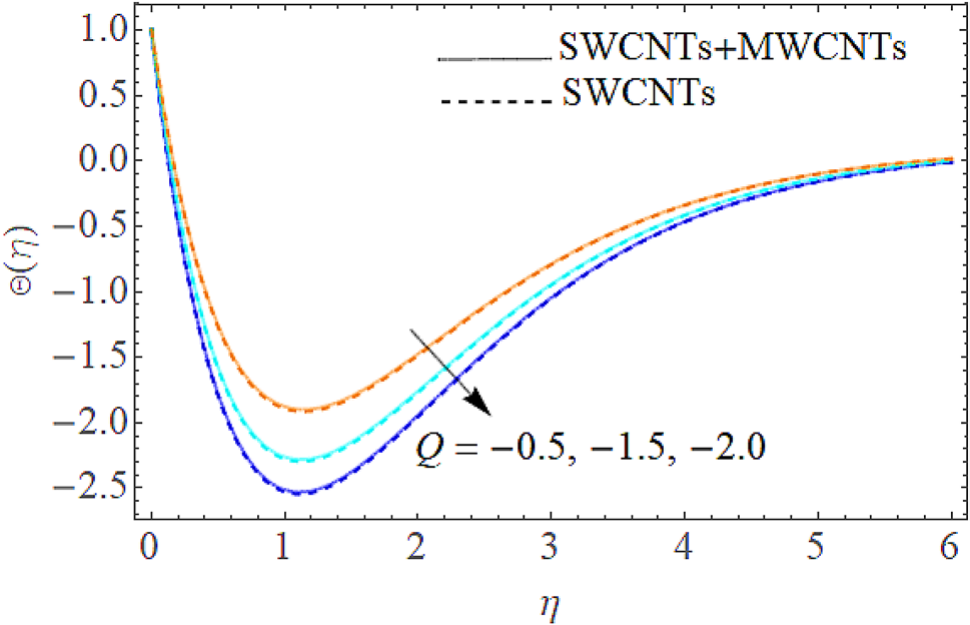
Impact of $Q$ on $\Theta (\eta )$.

The Eqs. (6) and (7) are explained by using the analytical technique HAM. The geometry of the model problem is shown in Fig. 1. The $h$ curves for both the velocity and thermal fields have been presented in Figs. 2 and 3. The parameters impact $\left( {E,\;M,\;Ec,\;\Pr ,\;k,\;Q} \right)$ on the hybrid nanofluid flow and temperature distribution has been displayed Figs. 4, 5, 6, 7, 8, 9, 10, 11 and 12. The default values of relevant parameters are used in the current section for mathematical calculation as $\left( {\,E = 2,\,\,M = 1,\,\,Ec = 9,\,\,\Pr = 21,\,\,k = 1.5} \right)F^{\prime}\left( \eta \right)$.The upsurge in the magnitude of $M$ strengthens the opposing force term as Lorentz force to diminish the $F^{\prime}\left( \eta \right)$ velocity profile as shown in Fig. 4. The obtained results show that $F^{\prime}\left( \eta \right)$ is decreasing with accumulative values of magnetic parameter. The strengthening in the values of $M$ enhancing the Lorentz force and this has happened due to the magnetic as well as electric fields in motion of electrically conducted fluid. Therefore, the weaker Lorentz force offers less opposing force to the transport phenomenon, that is why increase in $M$ corresponds to a weakening in the velocity of the Hybrid nanofluid. The effect of the Couple stress constraint $k$ over the velocity field is displayed in Fig. 5. The rising values of $k$ boosting the viscous forces and consequently the velocity field reduce.

The addition of the electric field $E$ with the magnetic field opposing the resistance against the fluid flow and displayed in Fig. 6. The electric field is in the addition while the magnetic field is in subtraction with the main equations. The addition of any term with the momentum equation, improving the momentum boundary layer. Therefore, the larger amount of the electric field improves the velocity. $\Pr$ (Prandtl number) effect over the thermal distribution is shown in Fig. 7. When using pure blood as the base fluid, it's worth noting that the Prandtl number of blood is 21, which is very high as compared to water and other common base fluids. Prandtl number is the ratio of momentum diffusivity to thermal diffusivity that’s why its larger values decline the temperature distribution. In fact, the thermal conductivity term is in the inverse relation to the Prandtl number and the greater value of $\Pr$ decline the thermal boundary layer. The ratio of the kinetic energy and fluid enthalpy is known as the Eckert number $Ec$ and the role of this number in the energy equation is displayed in Fig. 8. $Ec$ is used to illustrate the effect of self-warming of a liquid as a result of the dissipation term. The rising values of $Ec$ dominating the temperature profile. The growing amount of the magnetic parameter $M$ diminishes the magnitude of the liquid velocity and consequently the thermal boundary layer upsurges as shown in Fig. 9. The electric parameter $E$ impact over the thermal field is displayed in Fig. 10. Temperature is improved via higher valuation of $E$. Figure 11 shows the outputs of the parameter $Q$ on thermal field. The increasing value of the parameter $Q$ (source of heat) improving the temperature field and opposing effect observed using the concept heat sink as shown in Fig. 12.

### Entropy generation rate and Bejan number

**Figure 13:**
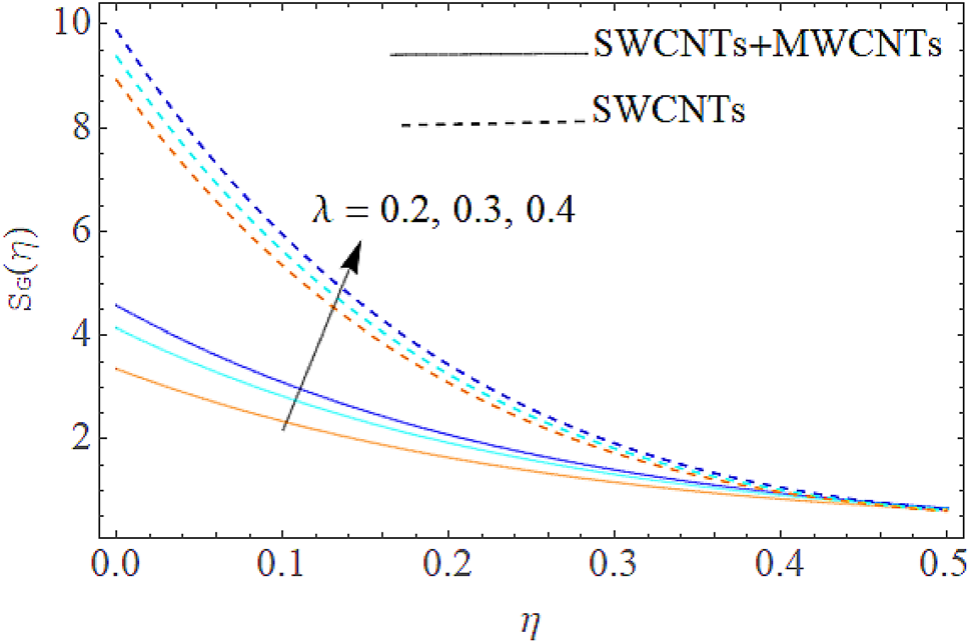
Outcome of $\lambda$ on $S_{G}$.

**Figure 14:**
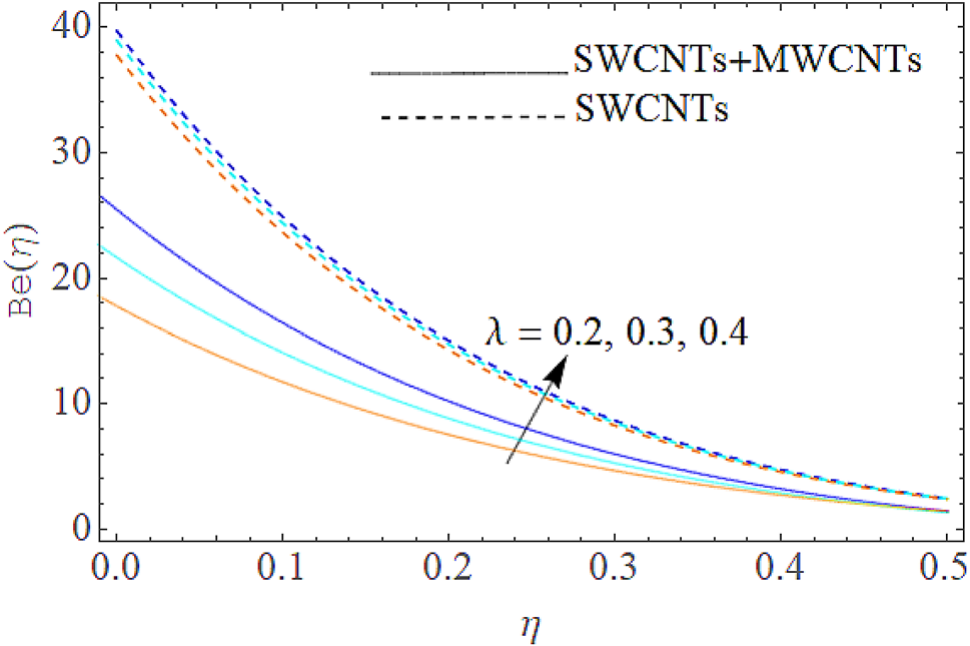
Influence of $\lambda$ on $Be$.

**Figure 15:**
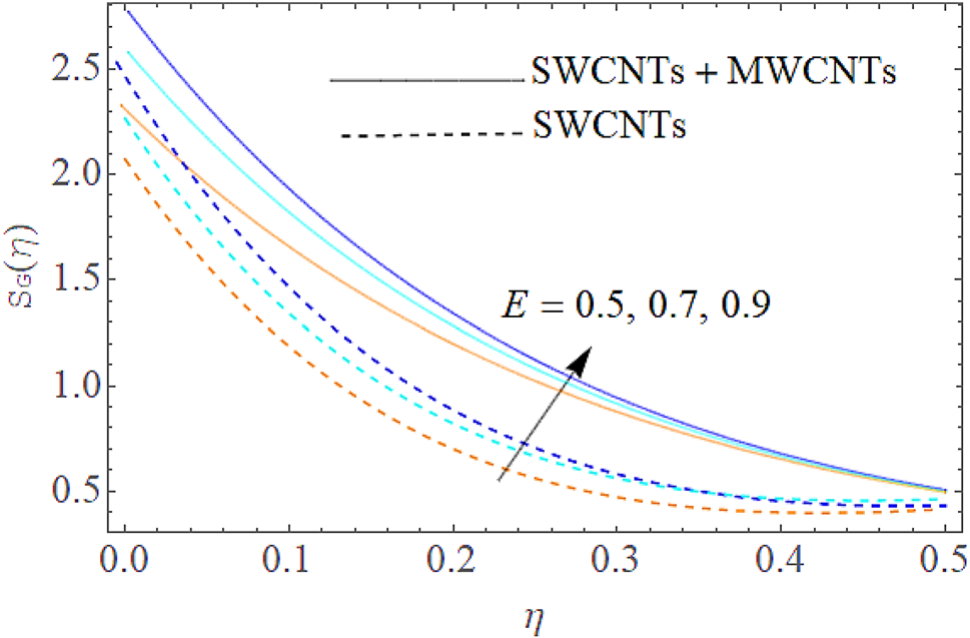
Outcome of $E$ on $S_{G}$.

**Figure 16:**
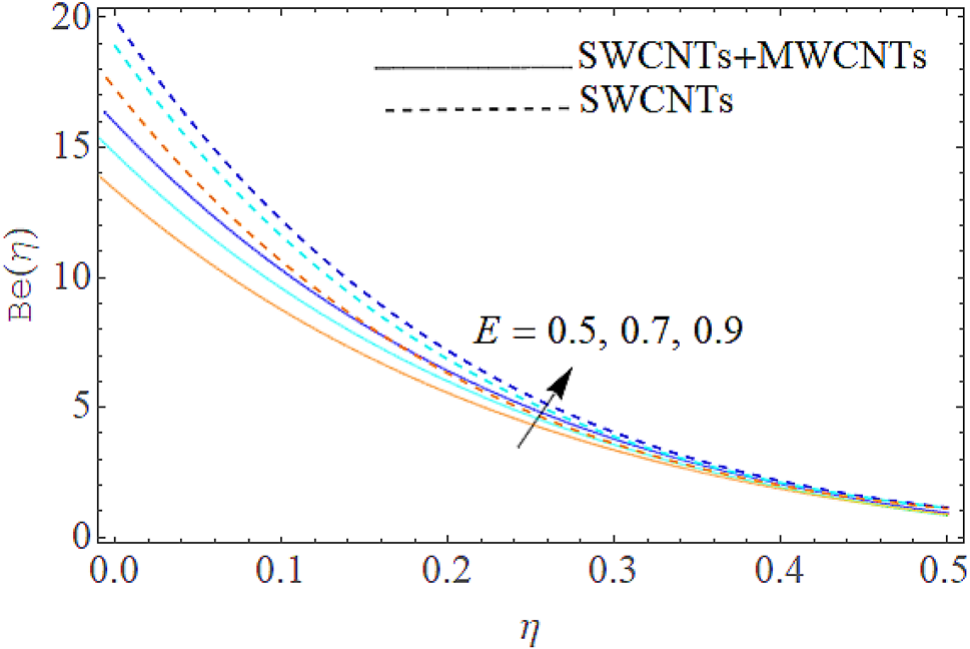
Influence of $E$ on $Be$.

**Figure 17:**
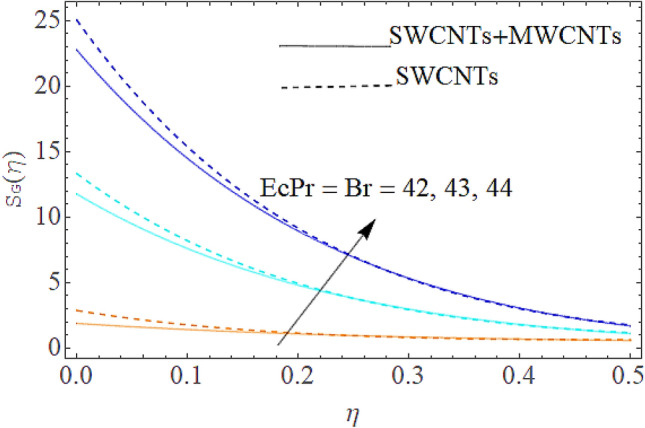
Impact of $Br$ on $S_{G}$.

**Figure 18:**
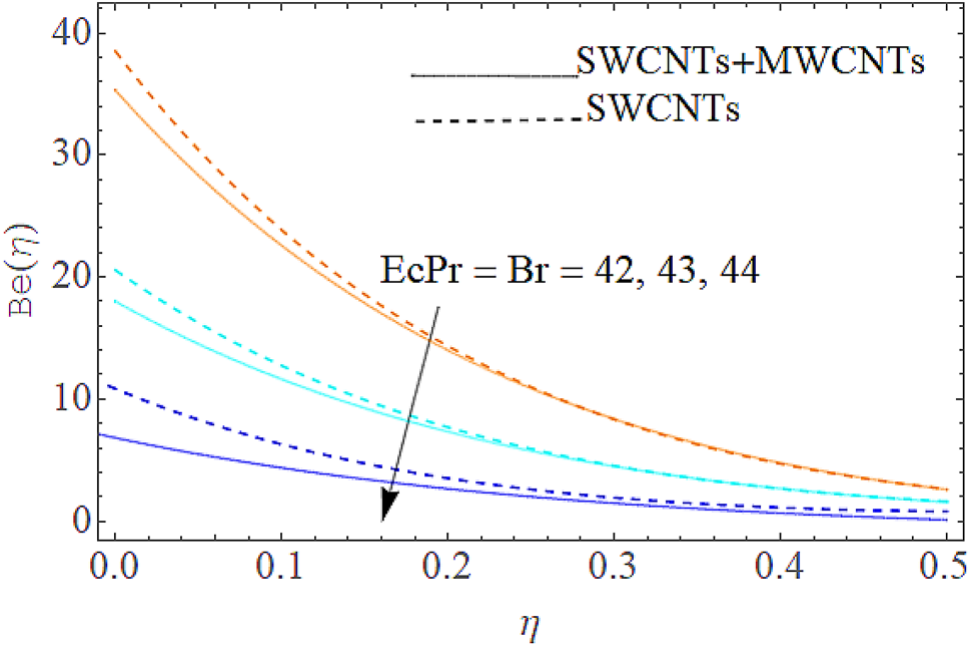
Outcome of $Br$ on $Be$.

**Figure 19:**
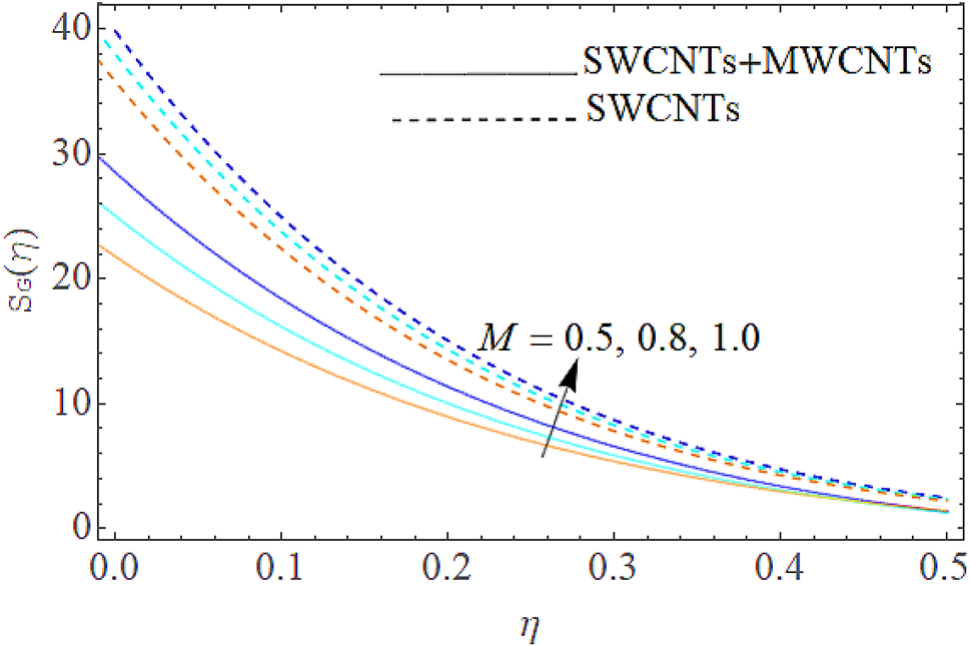
Outcome t of $M$ on $S_{G}$.

**Figure 20:**
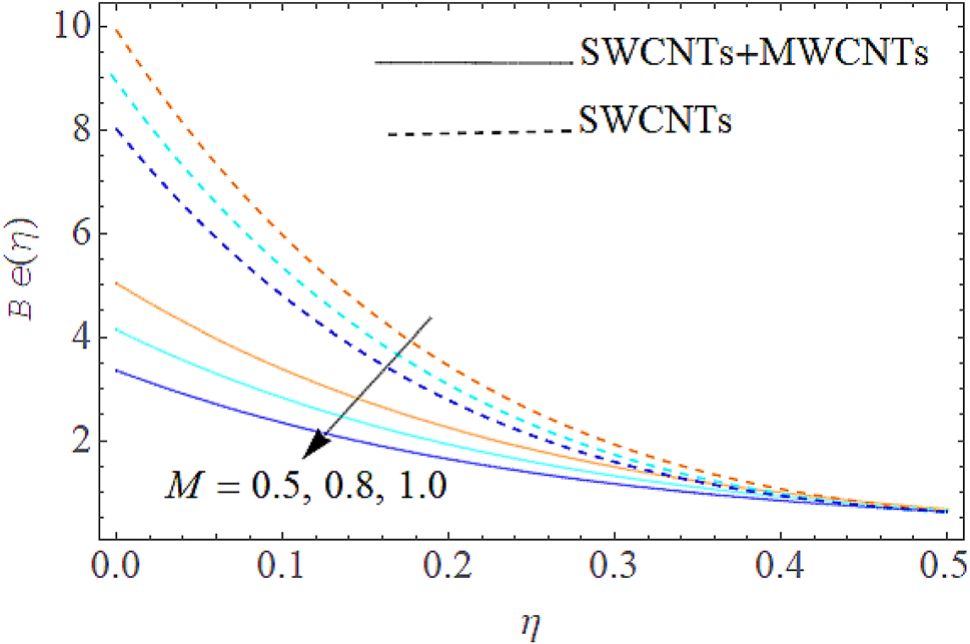
Influence of $M$ on $Be$.

**Figure 21:**
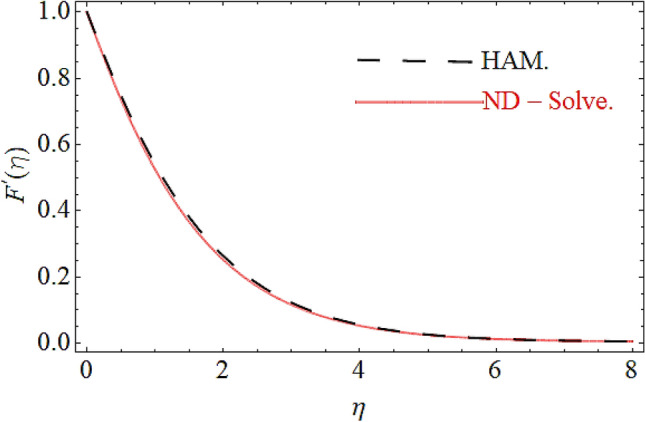
Comparison of HAM and ND-Solve in case of $F^{\prime}(\eta )$.

**Figure 22:**
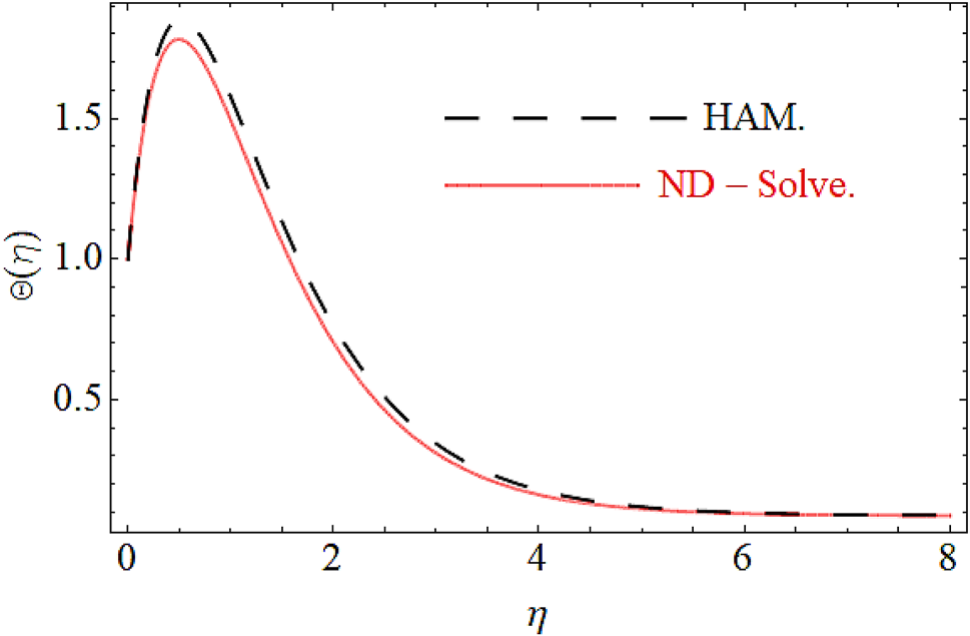
Comparison of HAM and ND-Solve in case of $\Theta (\eta )$.

**Figure 23:**
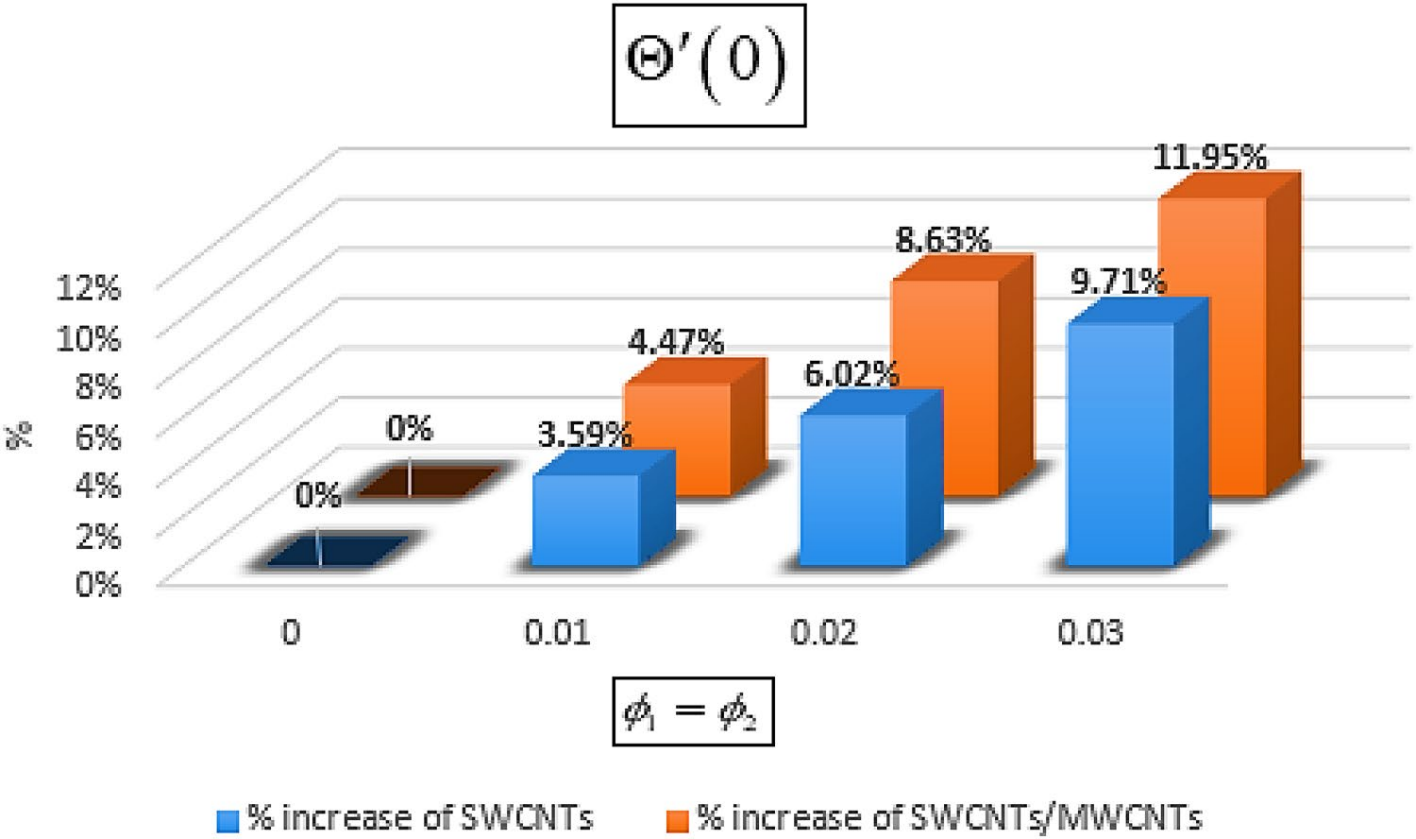
% enhancement in the heat transfer rate.

**Figure 24:**
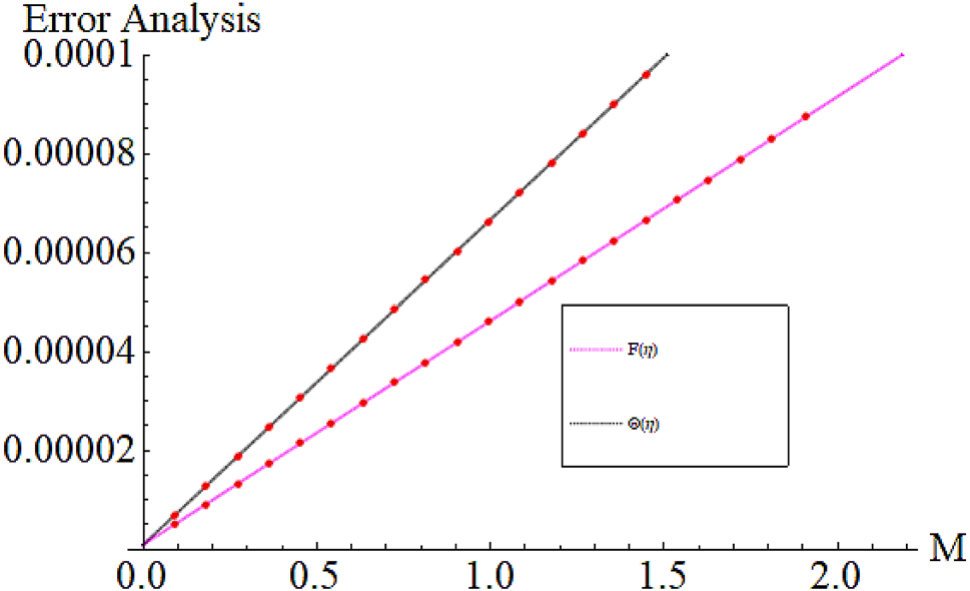
Parameter range $$M$$.

**Figure 25:**
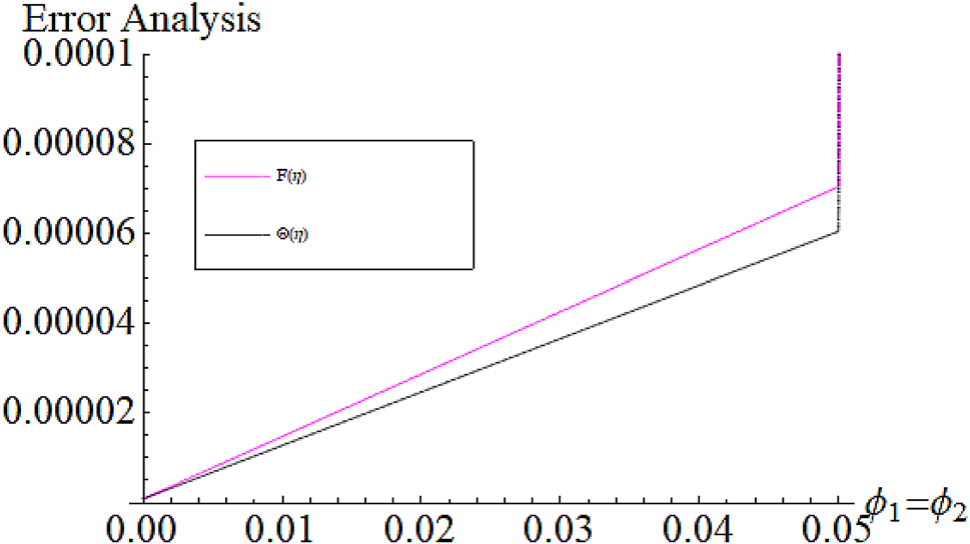
Parameter range $$\phi _{1} ,\phi _{2}$$.

**Figure 26:**
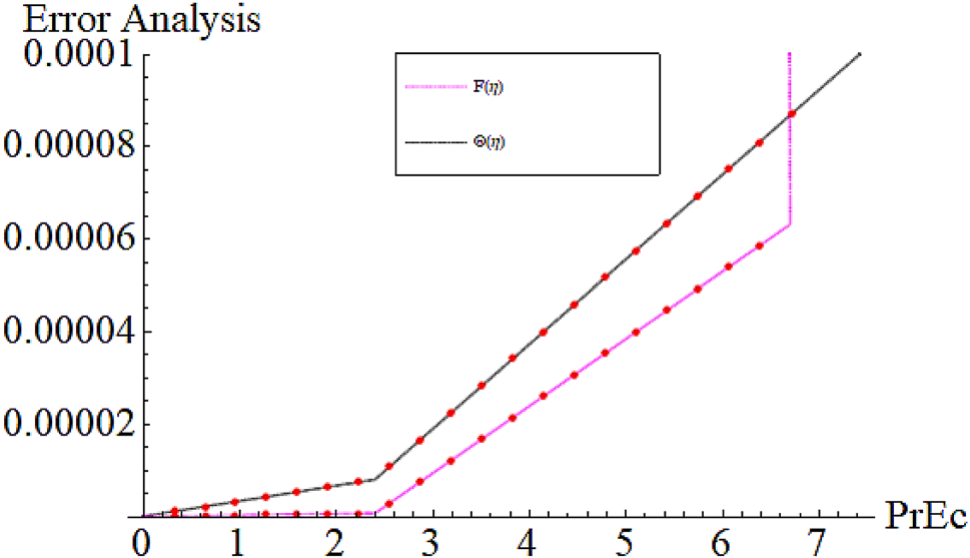
Parameter range $$Br = \Pr ,Ec$$.

From Figs. 13 and 14 we realize that $S_{G}$ and Bejan number upsurges with equivalent expansion in temperature difference factor correspondingly as displayed in these figures. Figures 15 and 16 have been drawn to indicate the consequences of the parameter $E$ on $S_{G}$ and $Be$ (Bejan number). Obviously, $S_{G}$ and Bejan number is boosted for electric field parameter. Figures 17 and 18 scrutinized the performance of Brinkman number on $S_{G}$ and Bejan number. Clearly $S_{G}$ is boost up via Brinkman number. The entropy rate rises for the larger values of $Br = \Pr Ec$. In fact, the thermal efficiency declines with the increasing amount of $Br$ while this effect is opposite in the irreversibility analysis $Be$. Bejan number decays from a physical perspective as the overall entropy production rate increases. Variation of $S_{G}$ and Bejan number versus magnetic factor $M$ is exposed in Figs. 19 and 20. Noticeably advanced magnetic parameter $M$ yields additional Lorentz forces which expands the opposition to liquid flow and therefore $S_{G}$ amplified. One can clearly find that for larger $M$ the Bejan number reduced. The comparison of HAM and Numerical ND-Solve method for the velocity and temperature profiles are displayed in Figs. 21 and 22. The closed agreement has been achieved from these two methods. The enhancement in the heat transfer rate is shown in Fig. 23. Excel software has been used to draw the Fig. 23 in which the % increase in the heat transfer rate has been calculated for the SWCNTs and Hybrid nanofluid. This figure shows that the heat transfer rate is frequently raises with the hybrid nanofluids as compared to the traditional fluids. As compared to conventional fluids, hybrid nanofluids have been shown to be more effective for heat transmission improvement. The parameters range for the proposed problem are shown in Figs. 24, 25 and 26.

### Table discussion

In this subsection various tables are constructed to exhibit the numerical influence of some physical factors for drag force and heat transfer rate. In Table 1 impact of mixed magnetic parameter $M$, couple stress parameter $k$, electric field parameter $E$ and $\phi _{2}$ upon skin friction are evaluated. The magnetic parameter $M$, couple stress parameter $k$ and volume fraction $\phi _{2}$ upsurge the drag force which enhance the cohesive forces among the blood particles and consequently the skin friction coefficient $Cf_{x}$ rises. The addition of the electric field $E$ with the magnetic field opposing the resistance against the fluid flow and therefore, the augmentation in the electric field $E$ reduces $Cf_{x}$. In Table 2 impact of Prandtl number $Pr$, magnetic parameter $M$, electric field parameter $E$, $Q$, $\phi _{2}$ and Eckert number $Ec$ upon Nusselt number are shown numerically. The increasing values of the parameters $M$, $E$, $Q$, $\phi _{2}$ and $Ec$ increasing the heat transfer rate and this effect for all the mentioned parameters is more effective using the hybrid nanofluid. Therefore, the normal temperature sustainability in blood is more effective through hybrid nanofluid. On the other hand the Prandtl number decreasing the heat transfer rate for its larger values. Table 3 shows the numerical comparison of old results with the recent result.

**Table 1:** Skin friction $C_{{fx}} Re_{x} ^{{0.5}}$ versus various parameters for the blood base hybrid nanofluid.

$M$	$\phi _{1} = \phi _{2}$	$k$	$E$	SWCNTs $F^{\prime\prime}\left( 0 \right)$	SWCNTs + MWCNTs $F^{\prime\prime}\left( 0 \right)$
0.0	0.0	0.1	0.1	1.23372	1.34584
0.2				1.41008	1.52120
0.4	0.0			1.73345	1.81567
	0.01			1.78456	1.88421
	0.02			1.85272	1.951534
	0.03			1.88121	2.012271
		0.2		1.942033	2.15314
		0.3		1.97624	2.202141
			0.2	1.72121	1.941124
			0.3	1.61043	1.9153123

**Table 2:** $\Theta ^{\prime}(0)$ versus various parameters for the blood base hybrid nanofluid.

$\phi _{1} = \phi _{2}$	$M$	$Q$	$E$	$Ec$	$\Pr$	SWCNTs $\Theta ^{\prime}\left( 0 \right)$	SWCNTs + MWCNTS $\Theta ^{\prime}\left( 0 \right)$
0.0	0.2	0.2	0.2	0.2	6.2	0.132456	0.240567
0.01						0.137214	0.251325
0.02						0.140431	0.261332
0.03						0.145323	0.269315
	0.3					0.203221	0.3141121
	0.4					0.244151	0.3152211
		0.3				0.3103251	0.4136752
		0.4				0.405104	0.51226132
			0.3			0.51230532	0.60135101
			0.4			0.610462	0.71567810
				0.3		0.721045	0.83118013
				0.4		0.811321	0.91908723
					6.3	0.710132	0.82121651
					6.7	0.612032	0.72314273

**Table 3:** Comparison with^41,42^ taking the similar parameters and excluding diverse constraints.

$\Pr$	Wang^42^	Golra and Sidawi^41^	Recent
6.2	1.2813	1.28135	1.281367
6.3	1.3120	1.31203	1.312042
6.4	1.5672	1.56721	1.567225

## Conclusions

The hybrid nanofluid flow over an extending surface is analyzed in this research. The couple stress nanofluid with solid materials of CNTs is considered, including Joule heating and viscous dissipation. The hybrid nanofluid is prepared due to the suspension of the solid nanoparticles of the SWCNTs and MWCNTs in pure blood. This mathematical model is an appropriate model for the biological advantages. The HAM solution of the modeled problem has been obtained. The impact of the embedded constraints over the hybrid nanofluid flow are drawn and discussed. The result indicates that hybrid nanofluids are the best thermal conductor and plays an important role in the circulation of blood in various parts of the human body. The stabilization of urine in ureters and so on is the applications of the hybrid nanofluid.

The main outputs are pointed out as:

- The heat absorption and omission constraint influences have been observed in the thermal field and this parameter has the tendency to retain the normal temperature in the case of the blood flow.
- The Prandtl number used for the blood is 21 and very large as compared to the other base fluids. These outcomes show that in case of the velocity of surface is larger than the velocity of the free stream, the reduced number rises with the rise of $\Pr$.
- The transverse Magnetic field $M$ declines the flow regime and $F^{\prime}\left( \eta \right)$ upsurges with the augmentation of $E$.
- The increasing values of the parameters $M$, $E$, $Q$, $\phi _{2}$ and $Ec$ increasing the heat transfer rate and this effect for all the mentioned parameters is more effective using the hybrid nanofluid. Therefore, the normal temperature sustainability in blood is more effective through hybrid nanofluid.
- The output shows that hybrid nanofluid are the most efficient sources to increase the heat transfer rate in the blood flow.
- 11.95% enhancement in the heat transfer rate has been observed at the 3% increase in the nanoparticle volume fraction.

## Data Availability

The data that support the findings of this study are available from the corresponding author upon reasonable request.

## List of symbols

*u*, *v*: Components of velocity (ms^−1^)
*B*_0_: Strength of magnetic field (NmA^−1^)
*E*_0_: Strength of electric field
*F*: Velocity profile dimensionless
*T*: Temperature of liquid (K)
*T*_*w*_: Temperature of surface (K)
T_∞_: Temperature of free surface (K)
*Q*_0_: Heat absorption/omission
*M*: Magnetic parameter
*k*: Couple stress parameter
*Q*: Heat source/sink parameter
Pr: Prandtl number
*E*: Electric field parameter
Ec: Eckert number
*q*_*w*_: Heat flux
N*u*: Nusselt number
C_*f*_: Skin friction coefficient
*S*_*G*_: Rate of entropy generation
*Br*: Brinkman number
*Be*: Bejan number
*b*: Stretching constant
(*C*_*p*_)_*f*_: Specific heat of base fluid (J/kgK)
*k*_*nf*_: Thermal conductivity (Wm^−1^ K^−1^

## Greek symbols

*μ*_*hnf*_: Hybrid nanofluid dynamic viscosity
*μ*_*f*_: Base fluid dynamic viscosity (mPa)
*σ*^*^: Electric conductivity
*ρ*_*nf*_: Nanofluid density (kgm^−3^)
*ρ*_*f*_: Base fluid density (kgm^−3^)
*η*: Similarity variable
*ϕ*1 and *ϕ*2: Volume fraction of *SWCNTs* and *MWCNTs* nanoparticles
$$L_{{\overset{\lower0.5em\hbox{$\smash{\scriptscriptstyle\frown}$}}{F} }} ,\;{\text{L}}_{{\overset{\lower0.5em\hbox{$\smash{\scriptscriptstyle\frown}$}}{\Theta } }}$$: Linear operators
*Θ*: Dimensional heat profile
*τ*_*w*_: Shear stress
*λ*: Temperature difference parameter
*λ*^*^: Mixed convection parameter
$\varepsilon _{m}^{t}$: The total residual error

## Subscripts

nf: Nanofluid
f: Base fluid
hnf: Hybrid nanofluid

## Abbreviations

HAM: Homotopy analysis method
SWCNT: Single wall carbon nanotube
MWCNT: Multi wall carbon nanotube
EMHD: Electromagnetic-hydrodynamics
CNTs: Carbon nanotubes

## References

[1] Choi SUS (1995). Enhancing thermal conductivity of fluids with nanoparticles. ASME Publ. Fed..

[2] Eastman JA, Choi SUS, Li S, Yu W, Thompson LJ (2001). Anomalously increased effective thermal conductivities of ethylene glycol-based nanofluids containing copper nanoparticles. Appl. Phys. Lett..

[3] Khan WA, Pop I (2010). Boundary-layer flow of a nanofluid past a stretching sheet. Int. J. Heat Mass Transf..

[4] Seth GS, Kumar B, Nandkeolyar R (2019). MHD mixed convection stagnation point flow of a micropolar nanofluid adjacent to stretching sheet: A revised model with successive linearization method. J. Nanofluids.

[5] Rashidi MM, Ghahremanian S, Toghraie D, Roy P (2020). Effect of solid surface structure on the condensation flow of Argon in rough nanochannels with different roughness geometries using molecular dynamics simulation. Int. Commun. Heat Mass Transf..

[6] Mansoury D, Doshmanziari FI, Rezaie S, Rashidi MM (2019). Effect of Al_2_O_3_/water nanofluid on performance of parallel flow heat exchangers. J. Therm. Anal. Calorim..

[7] Rashad AM, Rashidi MM, Lorenzini G, Ahmed SE, Aly AM (2017). Magnetic field and internal heat generation effects on the free convection in a rectangular cavity filled with a porous medium saturated with Cu–water nanofluid. Int. J. Heat Mass Transf..

[8] Sheikholeslami M (2019). New computational approach for exergy and entropy analysis of nanofluid under the impact of Lorentz force through a porous media. Comput. Methods Appl. Mech. Eng..

[9] Hatami M, Zhou J, Geng J, Song D, Jing D (2017). Optimization of a lid-driven T-shaped porous cavity to improve the nanofluids mixed convection heat transfer. J. Mol. Liq..

[10] Hatami M, Jing D (2017). Optimization of wavy direct absorber solar collector (WDASC) using Al_2_O_3_–water nanofluid and RSM analysis. Appl. Therm. Eng..

[11] Tang W, Hatami M, Zhou J, Jing D (2017). Natural convection heat transfer in a nanofluid-filled cavity with double sinusoidal wavy walls of various phase deviations. Int. J. Heat Mass Transf..

[12] Hatami M (2017). Nanoparticles migration around the heated cylinder during the RSM optimization of a wavy-wall enclosure. Adv. Powder Technol..

[13] Esfe MH, Esfandeh S, Rejvani M (2018). Modeling of thermal conductivity of MWCNT–SiO_2_ (30: 70%)/EG hybrid nanofluid, sensitivity analyzing and cost performance for industrial applications. J. Therm. Anal. Calorim..

[14] Moghadassi A, Ghomi E, Parvizian F (2015). A numerical study of water based Al_2_O_3_ and Al_2_O_3_–Cu hybrid nanofluid effect on forced convective heat transfer. Int. J. Therm. Sci..

[15] Mohebbi R, Izadi M, Delouei AA, Sajjadi H (2019). Effect of MWCNT Fe_3_O_4_/water hybrid nanofluid on the thermal performance of ribbed channel with apart sections of heating and cooling. J. Therm. Anal. Calorim..

[16] Afrand M, Toghraie D, Ruhani B (2016). Effects of temperature and nanoparticles concentration on rheological behavior of Fe_3_O_4_–Ag/EG hybrid nanofluid: An experimental study. Exp. Therm. Fluid Sci..

[17] Izadi M, Mohebbi R, Karimi D, Sheremet MA (2018). Numerical simulation of natural convection heat transfer inside a┴ shaped cavity filled by a MWCNT–Fe_3_O_4_/water hybrid nanofluids using LBM. Chem. Eng. Process. Process Intensif..

[18] Esfe MH, Arani AAA, Badi RS, Rejvani M (2018). Ann modeling, cost performance and sensitivity analyzing of thermal conductivity of DWCNT–SiO_2_/EG hybrid nanofluid for higher heat transfer. J. Therm. Anal. Calorim..

[19] Asadi A, Asadi M, Rezaniakolaei A, Rosendahl LA, Afrand M, Wongwises S (2018). Heat transfer efficiency of Al_2_O_3_–MWCNT/thermal oil hybrid nanofluid as a cooling fluid in thermal and energy management applications: An experimental and theoretical investigation. Int. J. Heat Mass Transf..

[20] Iqbal Z, Maraj E, Azhar E, Mehmood Z (2017). A novel development of hybrid (MoS_2_–SiO_2_/H_2_O) nanofluidic curvilinear transport and consequences for effectiveness of shape factors. J. Taiwan Inst. Chem. Eng..

[21] Gul T, Bilal M, Shuaib M, Mukhtar S, Thounthong P (2020). Thin film flow of the water-based carbon nanotubes hybrid nanofluid under the magnetic effects. Heat Transf..

[22] Sheremet MA, Cimpean DS, Pop I (2020). Thermo-gravitational convection of hybrid nanofluid in a porous chamber with a central heat-conducting body. Symmetry.

[23] Huang M, Borzoei H, Abdollahi A, Li Z, Karimipour A (2021). Effect of concentration and sedimentation on boiling heat transfer coefficient of GNPs-SiO_2_/deionized water hybrid nanofluid: An experimental investigation. Int. Commun. Heat Mass Transf..

[24] Pavlov KB (1974). Magnetohydrodynamic flow of an incompressible viscous fluid caused by deformation of a plane surface. Magn. Gidrodin..

[25] Sheikholeslami M, Abelman S, Ganji DD (2014). Numerical simulation of MHD nanofluid flow and heat transfer considering viscous dissipation. Int. J. Heat Mass Transf..

[26] Khan WA, Makinde OD (2014). MHD nanofluid bioconvection due to gyrotactic microorganisms over a convectively heat stretching sheet. Int. J. Therm. Sci..

[27] Hsiao K-L (2017). Micropolar nanofluid flow with MHD and viscous dissipation effects towards a stretching sheet with multimedia feature. Int. J. Heat Mass Transf..

[28] Krishna MV (2020). Heat transport on steady MHD flow of copper and alumina nanofluids past a stretching porous surface. Heat Transf. Asian Res..

[29] Hayat T, Khan SA, Alsaedi A, Fardoun HM (2020). Heat transportation in electro-magnetohydrodynamic flow of Darcy–Forchheimer viscous fluid with irreversibility analysis. Phys. Scr..

[30] Chaudhary S, Kanika KM (2020). Viscous dissipation and Joule heating in MHD Marangoni boundary layer flow and radiation heat transfer of Cu–water nanofluid along particle shapes over an exponential temperature. Int. J. Comput. Math..

[31] Eldabe NTM, Salwa MGE (1995). Heat transfer of MHD non-Newtonian Casson fluid flow between two rotating cylinders. J. Phys. Soc. Jpn..

[32] Nadeem S, Haq RU, Akbar NS, Khan ZH (2013). MHD three-dimensional Casson fluid flow past a porous linearly stretching sheet. Alex. Eng. J..

[33] Nandkeolyar R (2018). A numerical treatment of unsteady three-dimensional hydromagnetic flow of a Casson fluid with Hall and radiation effects. Results Phys..

[34] Usman M, Soomro FA, Haq RU, Wang W, Defterli O (2018). Thermal and velocity slip effects on Casson nanofluid flow over an inclined permeable stretching cylinder via collocation method. Int. J. Heat Mass Transf..

[35] Shah Z, Kumam P, Deebani W (2020). Radiative MHD Casson nanofluid flow with activation energy and chemical reaction over past nonlinearly stretching surface through entropy generation. Sci. Rep..

[36] Alkasasbeh H, Swalmeh M, Bani Saeed H, Al Faqih F, Talafha A (2020). Investigation on CNTs-water and human blood based Casson nanofluid flow over a stretching sheet under impact of magnetic field. Front. Heat Mass Transf. (FHMT).

[37] Dero S, Mohd Rohni A, Saaban A (2020). Effects of the viscous dissipation and chemical reaction on Casson nanofluid flow over the permeable stretching/shrinking sheet. Heat Transf..

[38] Liao SJ (2014). Advance in the Homotopy Analysis Method.

[39] Liao SJ (2003). On the analytic solution of magnetohydrodynamic flows of non-Newtonian fluids over a stretching sheet. J. Fluid Mech..

[40] Turkyilmazoglu M (2011). Numerical and analytical solutions for the flow and heat transfer near the equator of an MHD boundary layer over a porous rotating sphere. Int. J. Therm. Sci..

[41] Gorla RSR, Sidawi I (1994). Free convection on a vertical stretching surface with suction and blowing. Appl. Sci. Res..

[42] Wang CY (1989). Free convection on a vertical stretching surface. J. Appl. Math. Mech. (ZAMM).

